# Ubiquitous Expression of CUG or CAG Trinucleotide Repeat RNA Causes Common Morphological Defects in a *Drosophila* Model of RNA-Mediated Pathology

**DOI:** 10.1371/journal.pone.0038516

**Published:** 2012-06-08

**Authors:** Kynan T. Lawlor, Louise V. O’Keefe, Saumya E. Samaraweera, Clare L. van Eyk, Robert I. Richards

**Affiliations:** Discipline of Genetics, School of Molecular and Biomedical Science and Australian Research Council Special Research Centre for the Molecular Genetics of Development, The University of Adelaide, Adelaide, South Australia, Australia; University of Florida, United States of America

## Abstract

Expanded DNA repeat sequences are known to cause over 20 diseases, including Huntington’s disease, several types of spinocerebellar ataxia and myotonic dystrophy type 1 and 2. A shared genetic basis, and overlapping clinical features for some of these diseases, indicate that common pathways may contribute to pathology. Multiple mechanisms, mediated by both expanded homopolymeric proteins and expanded repeat RNA, have been identified by the use of model systems, that may account for shared pathology. The use of such animal models enables identification of distinct pathways and their ‘molecular hallmarks’ that can be used to determine the contribution of each pathway in human pathology. Here we characterise a tergite disruption phenotype in adult flies, caused by ubiquitous expression of either untranslated CUG or CAG expanded repeat RNA. Using the tergite phenotype as a quantitative trait we define a new genetic system in which to examine ‘hairpin’ repeat RNA-mediated cellular perturbation. Further experiments use this system to examine whether pathways involving Muscleblind sequestration or Dicer processing, which have been shown to mediate repeat RNA-mediated pathology in other model systems, contribute to cellular perturbation in this model.

## Introduction

The expansion of polymorphic repeat sequences (dynamic mutation) is responsible for over 20 human diseases, including Huntington’s disease (HD), myotonic dystrophy and several types of spinocerebellar ataxia (SCA) [1]–[3]. In all cases affected individuals inherit a repeat number that exceeds a pathogenic threshold. Increased disease severity and earlier age-of-onset are correlated with repeat length expansion, which occurs within successive generations [1]–[3]. Expansion is associated with loss of repeat-containing gene function and recessive inheritance in a few cases [4], [5], however the majority of diseases appear to be caused by dominant, repeat-mediated mechanisms. As well as a shared genetic basis, many diseases show similar clinical features, including late onset neurodegeneration and movement disorder, highlighting the possibility that common pathways contribute to pathology in most, if not all cases of dominantly inherited dynamic mutation [1]–[3], [6].

In diseases including HD and some types of SCA, expanded CAG trinucleotide repeats are found within a coding region, producing an expanded polyglutamine tract within the final protein [2]. Polyglutamine is able to cause pathology through multiple possible pathways, including gain-of-function interactions inherent to the expanded protein tract and alterations to normal protein function [3], [7], [8]. Nevertheless, polyglutamine protein-mediated pathology is not able to account for diseases where repeats are unable to encode polyglutamine, due to the repeat sequence composition and/or presence of the repeat tract within non-coding regions of the associated gene. This class of ‘untranslated’ repeat diseases share similar repeat sequences, pathogenic thresholds and clinical features with the ‘polyglutamine’ diseases and includes myotonic dystrophy type 1 (DM1) and 2 (DM2), Huntington’s disease-like-2 (HDL-2), fragile X tremor/ataxia syndrome (FXTAS) and SCA8, 10 and 12. In these diseases, gain-of-function properties of the expanded repeat RNA transcript appear to be responsible for dominant pathology [9]–[11]. Since repeat-containing RNA is present whether repeats are coding or non-coding, it may also represent a common contributor to all the dominant expanded repeat diseases. Studies have reported the ability of repeat RNA to cause pathology in animal and cellular model systems, via multiple, and likely distinct pathways [12]–[21].

Pathology due to expanded repeat RNA was first attributed in DM1, caused by a non-coding CUG repeat within the 3′UTR of the *dystrophia myotonica-protein kinase* (*DMPK*) gene, and DM2, caused by a non-coding CCUG repeat within an intron of *ZNF9* (now called *CNBP*) [22], [23]. Expanded repeat RNA transcripts form a ‘hairpin’ secondary structure, which is able to bind and sequester specific RNA-binding proteins [24]–[26]. The most extensively characterised of these is Muscleblind-like 1 (MBNL-1), which is involved in the regulation of alternative splicing [27], [28]. Sequestration of MBNL-1 leads to mis-splicing of certain transcripts and subsequent disease [29]. Pathology is associated with the formation of nuclear RNA foci that co-localise with MBNL-1 and may therefore be sites at which sequestration occurs [24], [30]–[32]. A number of other proteins have been identified that are bound or mis-regulated by repeat RNA, including CUG-binding protein (CUG-BP) [14], [33], Pur-alpha [34] and some heterogeneous nuclear ribonucleoproteins (hnRNPs) [35], [36], indicating a complex set of both common and repeat sequence specific interactions.

The presence of RNA foci, co-localisation of MBNL-1 with foci and dysregulation of alternative splicing have also been observed in either human tissue or animal models of the untranslated repeat diseases, DM1, DM2 [29], FXTAS [37], [38], SCA8 [39], [40], HDL-2 [41] and SCA10 [36]. Based on these hallmarks it is proposed that the sequestration of MBNL-1 and dysregulation of splicing may be a common contributor to pathology. Furthermore, RNA foci that co-localise with MBNL-1 have been observed in human HD cells [42], supporting a role for this pathway in the polyglutamine diseases, however evidence for perturbation of this pathway in human patients is limited.

Recent studies have identified other mechanisms, distinct from RNA-binding protein sequestration, through which repeat RNA may contribute to common pathology. We and others have reported evidence supporting a pathogenic role for bi-directional transcription, producing complementary repeat transcripts that hybridise to form double-stranded RNA [19], [20]. Pathology is modified by altering Dicer-2 levels and is associated with the formation of 21nt repeat RNAs, highlighting a role for small RNA processing pathways in disease [20]. Bi-directional transcription has been identified in a number of diseases and therefore this pathway may be a common contributor to dominant pathology [43]–[48].

Evidence for a repeat-mediated mechanism of non-ATG initiated translation, occurring in all three reading frames, highlights another alternative mechanism that may account for common pathology [49]. Combined with bi-directional repeat transcription this mechanism may produce multiple homopolymeric protein tracts that can contribute to pathology [49], [50]. The increasing number of pathogenic agents and pathways identified highlights the need for model systems in which to better define the contributors to pathology in each case.

Expanded repeat disease involves late onset neurodegeneration and loss of affected tissue, making access to samples and the detection of early changes that underlie pathology difficult. Human studies are therefore limited and animal models have been essential in investigating pathology. *Drosophila* is well established as a model for expanded repeat disease and a number of key pathways are conserved and have been identified or characterised in this system [7], [8], [13]–[15], [17], [19], [20], [39], [50], [51]. Repeats can be ectopically expressed in non-essential tissues such as the eye, allowing phenotypes involving cellular perturbation to be examined in an otherwise viable organism [52]. The *Drosophila* system is also amenable to the powerful approach of examining modifier genes, thus identifying genetic pathways required for pathology that will inform further studies in vertebrate models and human tissue.

We recently reported phenotypes, due to co-expression in the *Drosophila* eye or neurons, of complementary repeat transcripts that form double-stranded repeat RNA [20]. However, no consistent phenotypes were observed in the eye or neurons when each repeat sequence was ectopically expressed in the absence of a complementary transcript, thus acting only as a single-stranded ‘hairpin’ RNA [17], [20]. Here we characterise a morphological phenotype caused by ubiquitous expression of CUG or CAG repeat RNA-containing transgenes in *Drosophila* identifying a new system, independent of the *Drosophila* eye, in which to study common ‘hairpin’ repeat RNA-mediated pathology. Ubiquitous expression of either CUG or CAG repeat RNA disrupts the adult *Drosophila* dorsal abdominal tergites, possibly through an effect on the developing histoblast cells. Results indicate that the tergite phenotype is quantitative and dependent on repeat-transgene expression, providing a biological read-out of ‘hairpin’ RNA mediated pathology in this model system.

Further experiments examine whether pathways known to contribute to repeat RNA-mediated pathology are rate limiting for tergite disruption. Reducing levels of Mbl, the *Drosophila* ortholog of MBNL-1, does not enhance pathology and only modifies CUG-mediated phenotypes.

In contrast to recently reported double-stranded repeat RNA-mediated pathology [20], reducing Dicer-2 (Dcr-2) levels was not rate limiting for ‘hairpin’ RNA-mediated tergite disruption. However, reducing Dicer-1 (Dcr-1) levels gave opposing results for phenotypes caused by CAG compared to CUG repeat RNA, highlighting the possibility of a sequence-dependent role for this pathway. Finally, examining RNA localisation identified specific RNA foci formed due to CUG, but not CAG, RNA expression. Thus, in this system ‘hairpin’ RNA may contribute to pathology through multiple interactions.

## Results

### Ubiquitous CAG or CUG Repeat RNA Expression Perturbs Abdominal Developmental in *Drosophila*

We previously reported a system to ectopically express ‘hairpin’-forming CUG or CAG repeat RNA transcripts under UAS control in *Drosophila* [8], [17], [20]. Co-expression of both CUG and CAG complementary repeat-containing transgenes gave Dcr-2 dependent eye phenotypes, behavioural phenotypes, and changes in the miRNA profile [20]. In contrast, expression of either one of CUG or CAG repeat-containing transgenes, in the absence of a complementary transcript, gave transcriptional changes indicative of cellular perturbation, but no observable phenotypes with ectopic expression in the neurons or eye [17], [20]. The absence of an observable phenotype limits the ability to genetically examine the basis of cellular perturbation caused by CUG or CAG repeat ‘hairpin’ RNA.

In this study we therefore set out to identify morphological phenotypes caused by ectopic expression of either CUG or CAG expanded repeat RNA in *Drosophila*. Ectopic expression of CUG or CAG expanded repeat RNA was compared to an empty vector (EV) negative control as well as ectopic expression of expanded CAA repeat RNA, which unlike CAG or CUG, is not predicted to form a stable secondary structure [53]. Experiments were undertaken using the system that we have previously reported, whereby different untranslated repeat RNA sequences are expressed within the 3′ untranslated region of an RNA encoding a short, non-functional peptide [8], [17]. Independent transgenic lines were examined for each repeat sequence, with each line carrying four independent insertions of the transgene to generate high steady state levels of RNA [20]. Multiple independent transgenic insertions were initially generated for each construct (*rCUG_∼100_*_,_ *rCAG_∼100_* or *rCAA_∼100_*_,_ named alphabetically, ‘A’ through to ‘K’) and used to create lines carrying sets of 4 independent transgene insertions (*4xrCAG_∼100_*, *4xrCUG_∼100_* or *4xrCAA_∼100_*, named numerically, ‘line 1’ onwards) [20]. We have previously shown that, in the case of CUG or CAG expression, these lines give comparable levels of transgene expression, so that phenotypic outcomes can be compared in each case [17], [20]. CAA expression transgenes appear to be present at a lower steady state level [17], possibly due to their inability to form stable secondary structures. Since it is possible that greater steady state RNA levels may be an outcome, rather than a cause, of pathogenic RNA-protein interactions, these lines were included nonetheless, along with other controls to ensure that robust comparisons were made in each case.

Expanded repeat RNA transgenes, under UAS control, were expressed ubiquitously using the *da-GAL4* driver [54]. Progeny expressing expanded CUG (*4xrCUG_∼100_*), or CAG (*4xrCAG_∼100_*) repeat RNA showed a variable reduction in adult viability compared to each of the controls. At the standard culture temperature of 25°C, a significant reduction was observed in one of two independent *4xrCUG_∼100_* expressing lines compared to those expressing *4xrCAA_∼100_* and the EV control (Table S1A). One of two independent *4xrCAG_∼100_* expressing lines showed a significant reduction compared to *4xrCAA_∼100_* expressing progeny, but not the EV control (Table S1A). When grown at 29°C, giving increased GAL4 activity and hence increased repeat expression levels, complete lethality was observed in *4xrCUG_∼100_* and *4xrCAG_∼100_* expressing progeny but not in either *4xrCAA_∼100_* expressing progeny or EV controls (Table S1B). These results therefore demonstrate that ubiquitous expression of expanded CUG or CAG repeat RNA leads to pathology in this *Drosophila* model.

**Figure 1:**
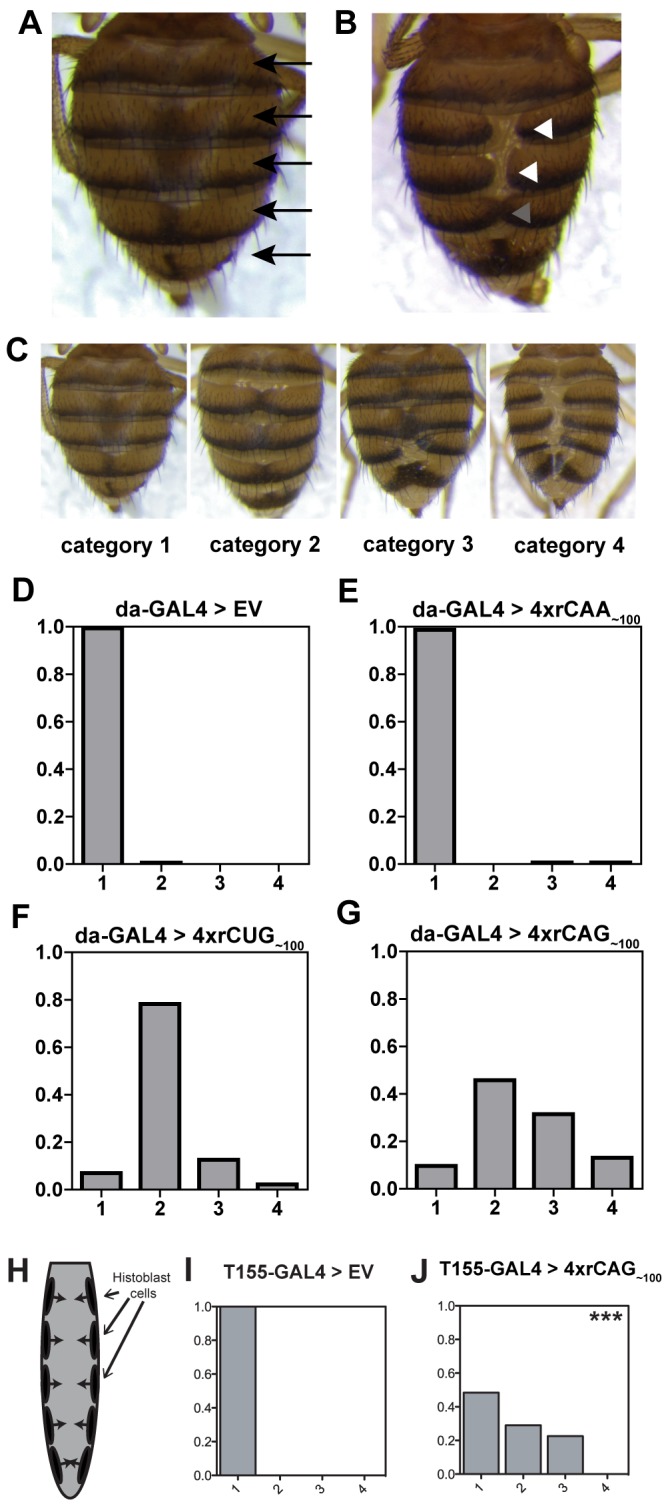
Tergite phenotype caused by ubiquitous expression of CAG or CUG repeat RNA in *Drosophila*. **A**, wild-type flies show a regular arrangement of tergite bands along the dorsal abdomen (arrows). **B**, An example of the disrupted phenotype, whereby tergites do not fuse at all (white arrowheads), or fuse only partially (grey arrowheads). **C**, Phenotype severity was scored on a scale of 1–4, images show typical examples from each category, where category 1 is like wildtype; category 2, tergites disrupted but not split; category 3, one tergite split; and category 4, two or more tergites split. **D-E**, Graphs showing the proportion of progeny within each scoring category. **D**, *da-GAL4* driven expression of the EV control line (n = 161) and, **E**, *da-GAL4* driven expression of *4xrCAA_∼100_* [line 1] (n = 148) gives no phenotype with almost all progeny like wild-type. **F**, *da-GAL4* driven expression of *4xrCUG_∼100_ [line 1]* (n = 271) or **G**, *4xrCAG_∼100_ [line 2]* (n = 343) gives a tergite disruption phenotype. **H,** Schematic (not to scale) showing the location of histoblast cells (black). Histoblasts proliferate and migrate to form the tergite bands (arrows). **I**, Expression within the histoblasts using *T155-GAL4* gives wild-type tergites in EV control progeny (n = 56). **J**, *T155-GAL4* expression of *4xrCAG_∼100_* [line 1] (n = 25) gives a mild tergite phenotype, ***p<0.001 comparing the proportion with a phenotype in **I** and **J**.

Closer physical examination of adult flies revealed a phenotype in viable progeny expressing expanded CUG or CAG repeat RNA at 25°C whereby the tergites of the dorsal abdomen were not correctly formed. In wild-type adults, the abdomen contains a series of regularly arranged tergites (Figure 1A), while in repeat expressing flies one or more of the tergite bands is split down the midline so that the two sides do not meet at all, or meet only partially (Figure 1B).

A strong phenotype was observed in both independent lines expressing 4x*rCAG_∼100_*, with semi-lethality and the strongest phenotype in *4xrCAG_∼100_ [line 1]*, and a moderate to strong phenotype in *4xrCAG_∼100_ [line 2]*. Both independent *4xrCUG_∼100_* lines gave a more moderate phenotype. This phenotype was not observed with expression of independent *4xrCAA_∼100_* lines, the EV control line, or in flies carrying each set of transgenes, in the absence of a GAL4 driver. Repeat expression via an independent ubiquitous driver, *Actin5C-GAL4*, gave an almost identical phenotype. In this case, the most severe phenotypes were observed in the same lines which gave the strongest *da-GAL4* phenotype (Table S2). Thus, ubiquitous expression of CUG or CAG repeat RNA gives rise to a specific morphological disrupted tergite phenotype.

To further confirm and characterise the disrupted tergite phenotype in each repeat expression line, a method was established to quantify phenotype severity. Newly eclosed adult female flies were individually scored and placed into one of four categories based on the severity of disruption and the number of tergites affected (See Methods) (Figure 1C). To give an overall measure of phenotype severity for each genotype, data was tallied to determine the proportion of progeny within each phenotype category (Figure 1D, E, F, G). Using this method, control progeny expressing either EV or each of two independent *4xrCAA_∼100_* transgenes were scored as having a phenotype at a frequency of only 0–2% (Figure 1D, E and Table S3). Similarly those carrying *da-GAL4* alone, or each UAS construct in the absence of GAL4 driver expression, were scored as having a phenotype in less than 1% of progeny (Table S3). Progeny expressing *4xrCAG_∼100_* had the most severe phenotype with independent transgenic lines showing at least 89% of progeny with a phenotype (Figure 1G and Figure S1). Independent lines expressing *4xrCUG_∼100_* showed a less severe phenotype, with at most 78% of progeny with a phenotype and a greater proportion within the mildest category, compared to *4xrCAG_∼100_* expression (Figure 1F and Figure S1). Decreased dosage of *rCAG_∼100,_* with ectopic expression from lines carrying two transgene copies, gave a more mild tergite phenotype than expression from the corresponding four transgene copy line (Figure S1). Thus, the phenotype is dependent on the number of transgene insertions being expressed, suggesting that increasing the dosage of available repeat-containing transcripts increases phenotype severity. Together, these results confirm that similar tergite disruption phenotypes are caused by the expression of CUG or CAG repeat-containing transcripts.

Although repeat RNA is ubiquitously expressed in this system, specific tergite disruption is observed, indicating that particular cells, or processes, may be sensitive to repeat RNA-mediated perturbation. The adult tergites are formed from the histoblast cells, a small population that is specified during early development and is located symmetrically, either side of the larvae [55]. During pupation these cells proliferate and migrate towards the midline, eventually giving rise to the tergite bands [56] (Figure 1H). We hypothesised that histoblast cells may be specifically perturbed by repeat expression, leading to the observed phenotype. To examine this possibility, repeats were expressed within histoblasts using the *T155-GAL4* driver [57],[58]. Expression from *4xrCAG_∼100_ [line 1]* gave a mild phenotype with *T155-GAL4*, while line 2 gave a phenotype in a very small, but significant proportion of progeny (Figure 1I&J and Figure S2). *4xrCUG_∼100_* expression with *T155-GAL4* did not give a significant phenotype in either independent transgenic line (Figure S2). The milder CAG phenotype, and absence of CUG phenotype, observed with this driver may be due to a lower level of expression than *da-GAL4,* or a requirement for repeat expression in neighbouring cell types to give a strong phenotype. Nonetheless, these results indicate that expression within histoblast cells is sufficient to cause tergite disruption, supporting the conclusion that cell specific perturbation is responsible for the phenotype.

### Tergite Disruption is not Enhanced by Reducing *Muscleblind* Levels

Expanded CUG or CAG repeat RNA expression in this *Drosophila* model leads to common perturbation of specific cells, giving a tergite disruption phenotype. This provides a quantitative biological read-out, and hence a model system to investigate whether genetically altering specific pathways modifies the observed repeat-mediated tergite phenotype. Using this approach, experiments examined whether pathways known to contribute to other forms of repeat RNA-mediated pathology also contribute to the disrupted tergite phenotype.

*Muscleblind (mbl)*, the *Drosophila* orthologue of MBNL-1, was chosen as an initial candidate, as previous studies in *Drosophila* have found that altering this gene can modify repeat RNA-mediated pathology in the eye [14], [15], [51]. To determine whether reducing *mbl* levels modifies tergite pathology, repeats were expressed with *da-GAL4* in progeny carrying one copy of the *mbl^E27^* mutant allele [27]. We hypothesized that if Mbl protein sequestration contributes to the phenotype, a further reduction in Mbl, in progeny heterozygous for the mutant allele, would lead to an enhanced phenotype.

**Figure 2:**
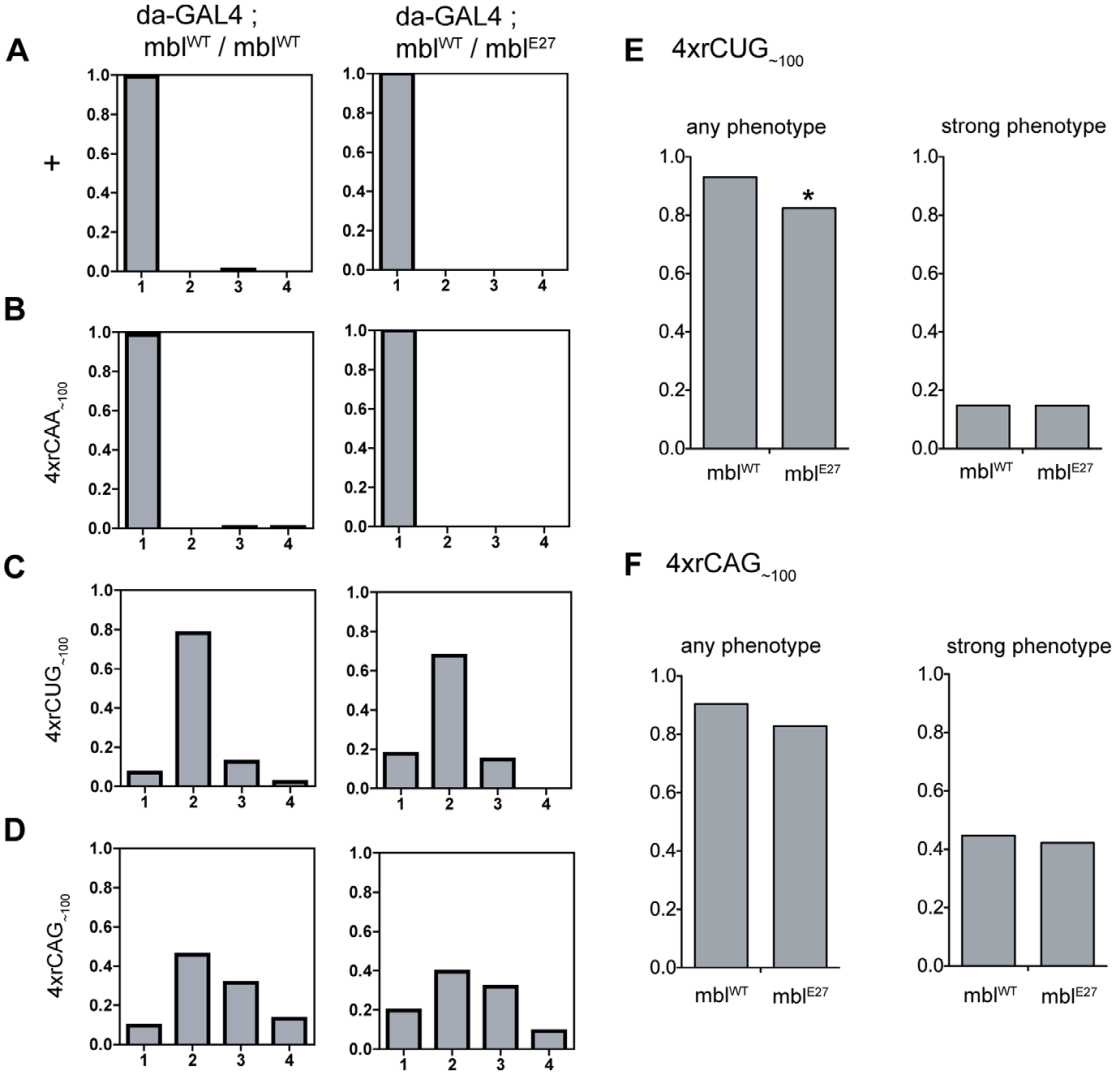
Reducing Mbl levels does not enhance the tergite phenotype. **A-D**, Proportion of progeny within each phenotype category when repeat expression is driven by *da-GAL4* alone (left column), or in the presence of the *mbl^E27^* mutant allele such that Mbl levels are reduced (right column). **A**, Wild-type control, **B**, *4xrCAA_∼100_ [line 1]*, **C**, *4xrCUG_∼100_ [line 1]*, **D**, *4xrCAG_∼100_ [line 2]*. **E, F**, Total proportion for each genotype that shows any phenotype (‘any phenotype’ - category 2, 3 and 4) and the proportion with a strong phenotype (‘strong phenotype’ – category 3 and 4). **E**, *4xrCUG_∼100_ [line 1]* shows a reduction in the proportion with any phenotype, but no change in the proportion with a strong phenotype with reduced Mbl levels. **F**, No significant effect was observed with *4xrCAG_∼100_ [line 2]*. Comparisons were made using Fisher’s exact test, with significant results indicated above the proportion, where *p<0.05.

The severity of the disrupted tergite phenotype was compared to determine whether repeat-mediated phenotypes were modified in progeny carrying one copy of *mbl^E27^*, compared to those with two wild-type *mbl* alleles (Figure 2A, B, C, D, S3 and Table S4). The proportion of progeny showing any phenotype (defined by progeny in all three phenotype categories) and the proportion showing a strong phenotype only (defined by progeny in the two most severe categories), were compared between genotypes (Figure 2E, F and Table S7). Statistical analysis revealed a significant (Fisher’s exact test) decrease in the proportion that show any phenotype with expression from two independent *4xrCUG_∼100_* lines in the presence of *mbl^E27^* (Figure 2E and S3). However, no change was observed when comparing the proportion within the strongest two categories only, perhaps indicating that only those with the weakest phenotype are suppressed (Figure 2E and S3). Expression of *4xrCAG_∼100_* in the presence of *mbl^E27^* led to no significant change in phenotype proportion compared to expression of *4xrCAG_∼100_* alone (Figure 2F and S3). Thus reducing Mbl levels did not enhance the tergite phenotype caused by either CUG or CAG repeat expression in this *Drosophila* system. Instead, suppression was observed that appeared to be specific to the phenotype caused by CUG expression only. Flies homozygous for *mbl^E27^* were not viable and thus it was not possible to examine the effect of complete Mbl loss-of-function in this system. However, these results do not support a role for Mbl sequestration as a common contributor to CUG or CAG RNA-mediated tergite phenotypes.

### Reduced Dicer-2 Processing is not Rate Limiting for the Tergite Phenotype

Previously we reported that co-expression of complementary CAG and CUG repeat RNA transcripts leads to pathology that is suppressed by reducing Dcr-2 levels [20]. Based on this evidence, and the presence of 21nt repeat RNAs, it is proposed that complementary repeats form double-stranded repeat RNA that is processed by Dicer enzymes [20]. Pathology is associated with altered miRNA profiles, indicating that double-stranded repeat RNA may perturb normal Dicer processing pathways [20]. Single-stranded ‘hairpin’ RNA has also been reported to be a substrate for Dicer processing, and therefore could potentially perturb Dicer processing pathways in a similar manner [59]. Ectopic expression of CUG or CAG repeat transgenes separately does not lead to a phenotype in the eye, such that modification by reducing Dicer processing could not be tested [20], [17]. However, as ubiquituous expression of each transgene gives tergite disruption, this phenotype can be used to genetically examine the role of Dicer processing in cellular perturbation caused by single-stranded CUG or CAG ‘hairpin’ repeat RNA, in the absence of a complementary transcript.

**Figure 3:**
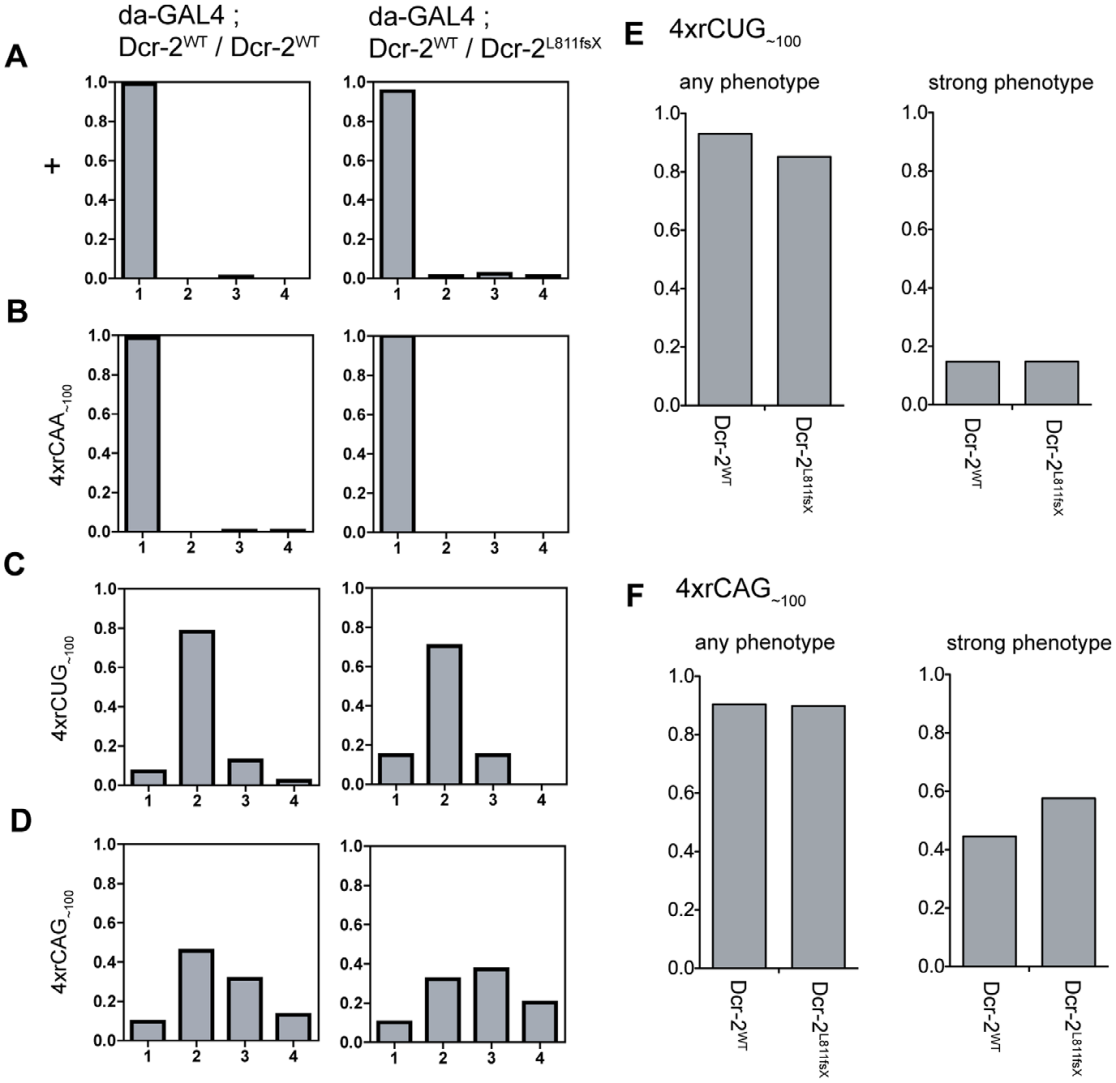
Reducing Dcr-2 levels is not rate limiting for the tergite phenotype. **A-D**, Proportion of progeny within each phenotype category when repeat expression is driven by *da-GAL4* alone (left column), or in the presence of the *Dcr-2^L811fsX^* mutant allele such that Dicer-2 levels are reduced (right column). **A**, Wild-type control, **B**, *4xrCAA_∼100_ [line 1]*, **C**, *4xrCUG_∼100_ [line 1]*, **D**, *4xrCAG_∼100_ [line 2]*. **E, F**, Total proportion for each genotype that shows any phenotype (‘any phenotype’ - category 2, 3 and 4) and the proportion with a strong phenotype (‘strong phenotype’ – category 3 and 4). **E**, *4xrCUG_∼100_ [line 1]*, **F**, *4xrCAG_∼100_ [line 2]*. None of the observed changes were statistically significant (Fisher’s exact test).

Experiments first examined whether reducing Dcr-2 levels can modify the tergite phenotype. Each of the expanded repeat RNA expression lines, *4xrCAG_∼100_*, *4xrCUG_∼100_* and *4xrCAA_∼100,_* were ubiquitously expressed via *da-GAL4* in the presence of one copy of the *Dcr-2^L811fsX^* loss-of-function mutant allele (Figure 3A, B, C, D, S4 and Table S5). If Dcr-2 function is rate limiting for the phenotype then this reduction in Dcr-2 levels would be expected to result in suppression, as was previously observed with complementary repeat RNA expression [20]. In independent lines, *4xrCUG_∼100_* and *4xrCAG_∼100_* expression in the presence of the *Dcr-2^L811fsX^* mutant allele did not give a significantly different phenotype severity compared to repeat RNA expression in a wild-type *Dcr-2* background (Figure 3E, F, S4 and Table S7). Thus, a reduction in Dcr-2 levels is not rate limiting for CAG or CUG repeat RNA-mediated tergite disruption. These results support tergite disruption being caused by a mechanism that is distinct to that responsible for Dcr-2 dependent double-stranded repeat RNA pathology.

### Reduced Dicer-1 Processing can have Opposing Effects on CUG or CAG RNA-mediated Tergite Phenotypes

**Figure 4:**
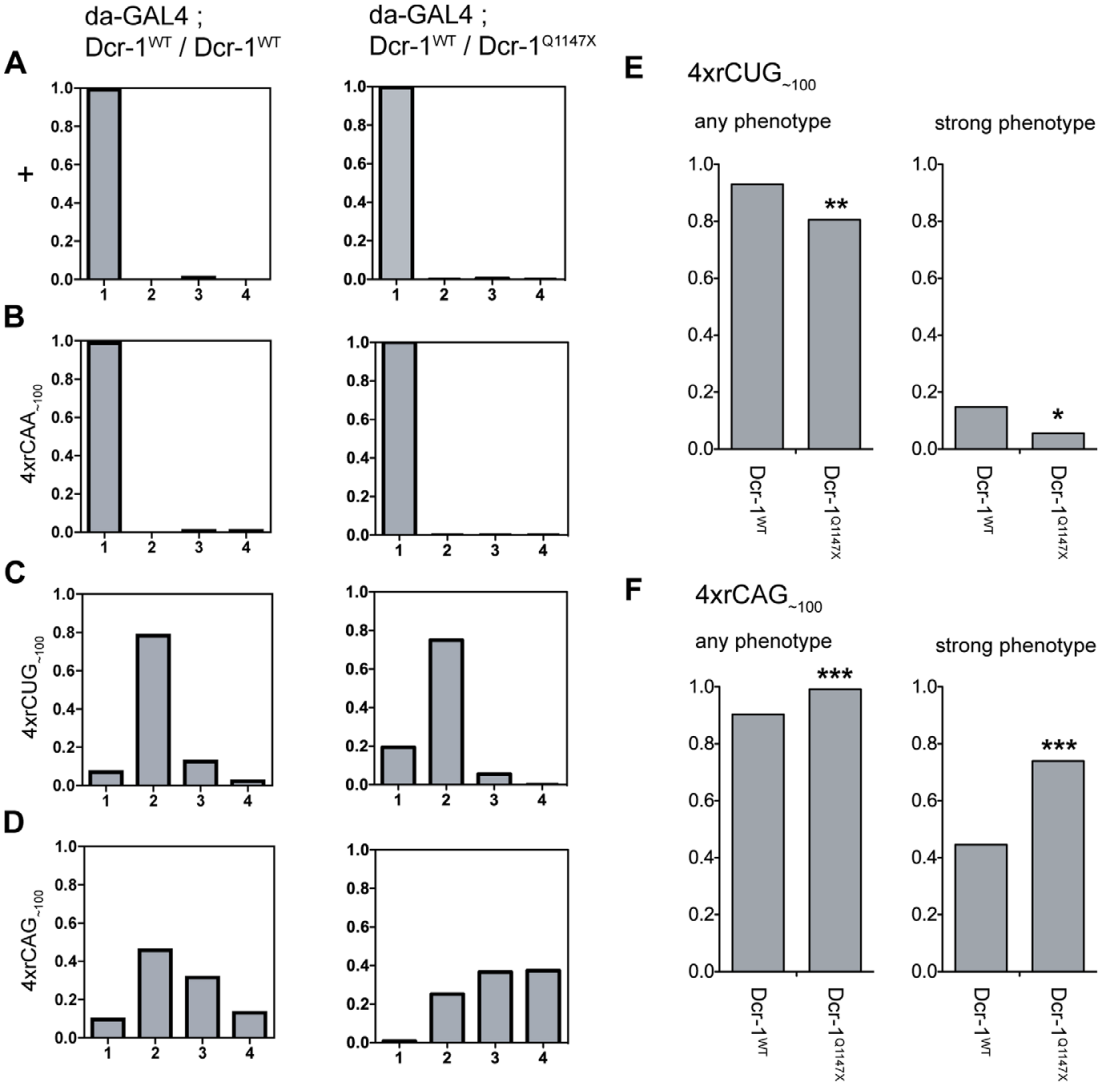
Reduced Dicer-1 processing can have opposing effects on CUG or CAG RNA-mediated tergite phenotypes. **A-D**, Proportion of progeny within each phenotype category for expression with *da-GAL4* alone (left column), or in the presence of the *Dcr-1^Q1147X^* mutant allele such that Dcr-1 levels are reduced (right column). **A**, wildtype control, **B**, *4xrCAA_∼100_ [line 1],* **C**, *4xrCUG_∼100_ [line 1],* **D**, *4xrCAG_∼100_ [line 2].* **E, F**, Total proportion for each genotype that shows any phenotype (‘any phenotype’ - category 2, 3 and 4) and the proportion with a strong phenotype (‘strong phenotype’ – category 3 and 4). **E**, *4xrCUG_∼100_ [line 1]* shows a suppression for both measures. **F**, *4xrCAG_∼100_* [line 2] shows an enhancement for both measures. Comparisons were made between the populations using Fisher’s exact test, with significant results indicated above the proportion, where *p<0.05, **p<0.01 and ***p<0.001.

In *Drosophila,* Dcr-1 and Dcr-2 have distinct roles in small RNA biogenesis and therefore may have different preferences for hairpin-forming repeat RNA [60]. Thus, the ability of reduced Dcr-1 levels to modify tergite pathology was also examined. Repeat constructs were ubiquitously expressed using *da-GAL4* in the presence of one copy of the *Dcr-1^Q1147X^* mutant allele and phenotype severity examined (Figure 4A-D, S5 and Table S6). In one of two lines expressing *4xrCUG_∼100_* in the presence of *Dcr-1^Q1147X^* the proportion of progeny with any phenotype, and those with a strong phenotype, was significantly reduced (Figure 4E, S5 and Table S7). A second *4xrCUG_∼100_* line gave a reduction in the proportion of progeny with any phenotype, however this was not statistically significant (Figure S5, Table S7). In contrast, one of two *4xrCAG_∼100_* lines showed a more severe phenotype in the presence of *Dcr-1^Q1147X^* than when the repeat was expressed alone, with both the proportion showing any phenotype, and a strong phenotype, significantly increased (Figure 4F and S5). It was not possible to confirm this finding in an independent line, as the second four transgene copy expression line already showed a strong phenotype and reduction in viability, giving insufficient progeny for analysis (Table S6). Results indicate that reducing Dcr-1 levels may have opposing effects on CUG or CAG mediated phenotypes in this system. A further reduction in Dcr-1 function may cause stronger effects, providing further evidence for this effect, however, flies homozygous for the *Dcr-1^Q1147X^* allele are not viable and the involvement of this protein in small RNA processing limits the feasibility of using RNAi methods. Nonetheless, our results do not support a simple model whereby Dcr-1 processing is rate-limiting for the common pathway leading to tergite disruption.

### Ubiquitous CUG, but not CAG, Repeat RNA Expression Gives Nuclear RNA Foci in Specific Cells

**Figure 5:**
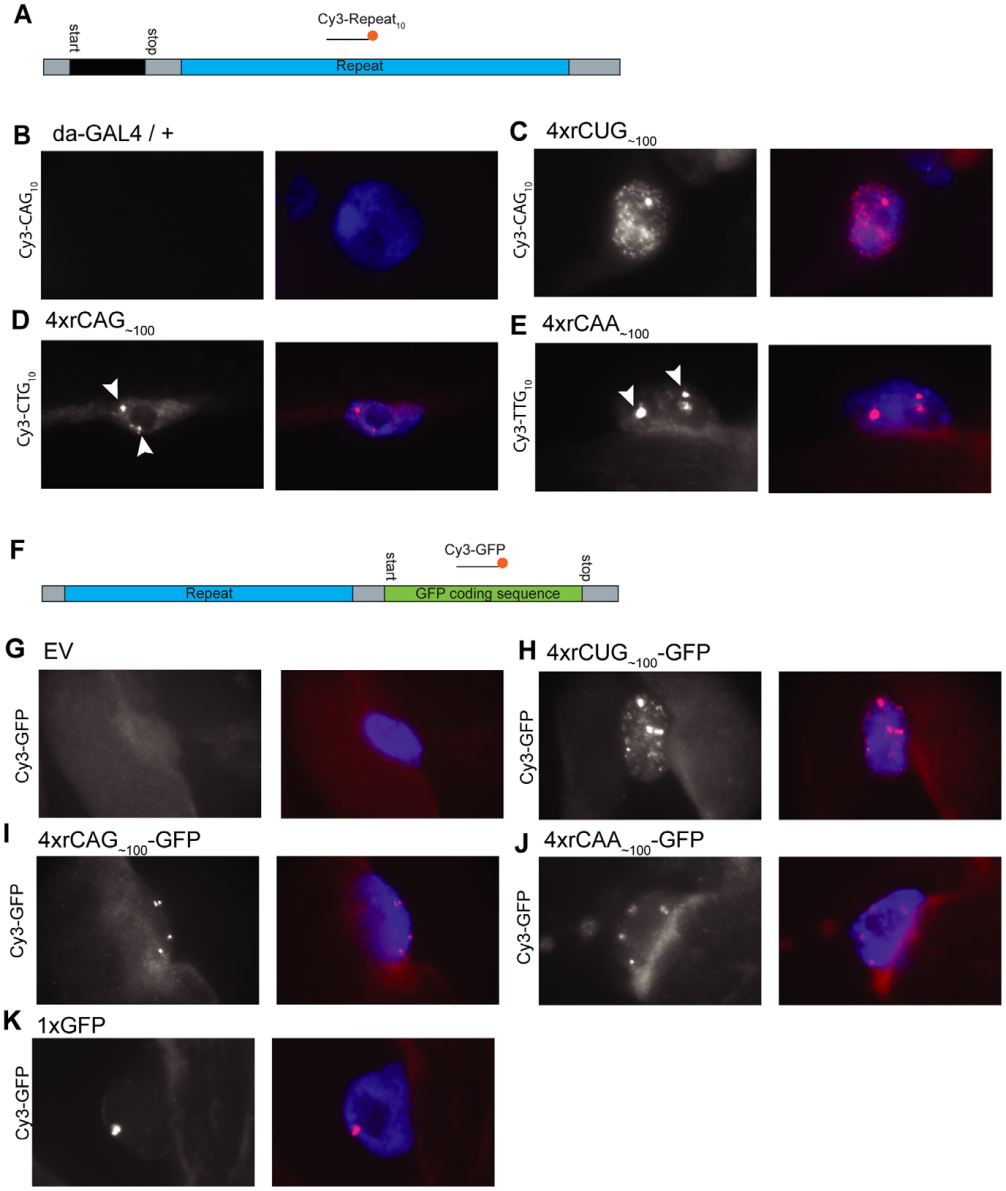
Ubiquitous CUG and CAG RNA repeat expression leads to distinct localisation in muscle nuclei. Images from cryosections hybridised with fluorescent probes to detect repeat RNA. Images are representative of observations from multiple animals and independent transgenic lines for each repeat. **A**, Schematic of the repeat expression construct, indicating the location of the repeat and probe. **B-E**, Repeat transcripts detected with a complementary repeat probe. **B**, Progeny carrying the *da-GAL4* driver alone show no signal in the nucleus. **C**, Expression of *4xrCUG_∼100_ [line 2]* leads to multiple foci throughout the nucleus. **D, E**, Expression of *4xrCAG_∼100_ [line 1]*, or *4xrCAA_∼100_ [line 1]* leads to between one and four sites of RNA concentration (arrowheads). **F**, Schematic of the construct giving repeat expression within the context of the GFP transcript, in this case RNA is detected using a probe against the GFP sequence. **G-K**, Repeat RNA localisation when expressed within a GFP transcript. **G**, No signal is observed using the GFP complementary probe against control EV progeny. **H**, *4xrCUG_∼100_-GFP* expression leads to a similar pattern of foci as in **C**. **I, J**, Expression of 4x*rCAG_∼100_-GFP* or *4xrCAA_∼100_-GFP* leads to a similar pattern as in **D** and **E**. **K**, Expression of a single copy of GFP, not containing a repeat, leads to the formation of a single similar site of RNA concentration.

A hallmark of pathology associated with ‘hairpin’ repeat RNA is the formation of nuclear repeat RNA foci [61]. Thus RNA localisation was examined to determine whether each of the repeat-containing transgenes being expressed in our system is able to form nuclear RNA foci. Expanded repeat RNA was visualised in cryo-sections of whole larvae by *in situ* hybridisation with a fluorescently labelled probe complementary to the repeat sequence (Figure 5). With da-GAL4 driven *4xrCUG_∼100_* expression, many nuclei contained up to four sites of RNA accumulation, possibly related to the sites of transcription for each transgene (data not shown). However, in a specific subset of cells, identified by morphology as larval muscle cells, multiple speckled foci were observed throughout the nucleus (Figure 5C). Identical staining was observed in independent samples and in independent transgenic lines (Figure S6). Furthermore, no signal was observed in lines carrying the repeat expression transgene but no GAL4 driver, confirming that RNA foci are dependent on transgene expression (Figure S6). *4xrCAG_∼100_* expression from independent transgenic lines gave up to four sites of RNA accumulation in all cell types, with multiple speckled foci not observed in muscle cells (Figure 5D, S7). Unexpectedly *4xrCAA_∼100_* expression gave a staining pattern almost identical to that observed with *4xrCAG_∼100_*. Up to four sites of RNA accumulation were observed in both muscle and non-muscle nuclei from independent transgenic lines expressing *4xrCAA_∼100_*, suggesting that sites of RNA accumulation are not ‘hairpin’-specific (Figure 5E, S8). These sites may be the sites of transcription, or processing, of the repeat containing transgenes, as the one to four sites observed correlate with the four transgenes being expressed. In each case, no staining was observed in nuclei carrying the transgene, but no GAL4 driver, suggesting that the observations are dependent on the transgene being transcribed (Figure S6, S7, S8).

RNA localisation results were confirmed by expressing each expanded repeat RNA sequence within the 5′UTR of the transcript encoding green fluorescent protein (GFP). In this case a probe complementary to the GFP sequence was used (Figure 5F), eliminating any effects specific to the binding dynamics of each repeat sequence. Lines carrying four transgenes were generated for each expanded repeat sequence: *4xrCUG_∼100_-GFP, 4xrCAG_∼100_-GFP* and *4xrCAA_∼100_-GFP*. Identical results were obtained to those observed initially, with *4xrCUG_∼100_*-GFP expression giving multiple foci within muscle cells (Figure 5H). However, *4xrCAG_∼100_*-GFP and *4xrCAA_∼100_-GFP* expression within a GFP encoding transcript gave only one to four sites of RNA concentration in all cell types (Figure 5I, J). Although each of the lines carried four independent transgene insertions, the number of sites of RNA concentration observed was between one and four for each cell. It is possible that the variation in this number is dependent on the focal plane observed for any given cell. In order to confirm this, a control line expressing a single GFP transgene, with no repeat sequence was examined. In this case nuclei showed a single site of RNA concentration in all cells, indicating that these RNA localisation experiments are detecting sites of transgene expression (Figure 5K).

In this model, ubiquitously expressed CUG, but not CAG or CAA-containing transcripts are able to undergo interactions sufficient to result in the formation of nuclear foci in specific cells. Thus, the mechanism that gives rise to cell-specific foci appears to be specific to CUG, but not CAG repeat RNA ‘hairpins’ in this system.

## Discussion

Recent evidence suggests that repeat RNA transcripts may contribute to dominantly inherited human pathology through multiple pathways [19], [20]. However, the specific mechanisms involved, particularly the way in which different repeat sequences give rise to similar pathology, is not fully understood. In this study we characterise a new system in which to study common CUG or CAG repeat RNA-mediated pathology. Reduced viability and a disrupted tergite phenotype were observed with ubiquitous expression of ‘hairpin’-forming CUG or CAG repeat RNA in this *Drosophila* model. Expression of either ‘hairpin’ repeat sequence gives similar tergite disruption, likely involving the same cell type, suggesting that a common pathway is responsible for cellular perturbation. In contrast, co-expressing both complementary repeat sequences in the same *Drosophila* system, to give double-stranded repeat RNA led to complete lethality [20]. Thus, single-stranded hairpin RNA may make a milder, more specific contribution to pathology, perturbing a particular pathway or cellular process and giving rise to susceptibility in certain cell types only. In support of this, expression of repeat RNA transcripts in histoblast cells was sufficient to cause mild tergite pathology.

Repeat RNA expression phenotypes observed in this study are not expected to mirror human pathology, but rather provide a tissue specific biological read-out of repeat-mediated cellular perturbation. The *Drosophila* eye has been used in a similar manner to show that key disease mechanisms identified in vertebrates appear to be conserved in flies [7], [8], [13]–[15]. The tergite phenotype provides an independent system in which to explore repeat RNA-mediated pathology. As distinct cell types, and biological processes, are predominant in each model tissue, an independent system may extend our knowledge of the genetic pathways that may contribute to pathology in human disease. The nature of the tergite phenotype itself suggests that processes involving cytoskeletal regulation, required for histoblast migration and intercalation, may be candidate pathogenic pathways [56]. The cytoskeleton is important for neuronal function and transcriptional changes in genes involved in cytoskeletal regulation were previously identified in microarray studies of neuronal repeat RNA expression in *Drosophila* [17]. While perturbation in neurons likely gives a different cell biological outcome than in histoblasts, the molecular pathways involved may be the same in each case. Thus, this *Drosophila* system provides a way to study these pathways using a quantitative morphological read-out, that would likely not be observed with neuronal perturbation. Furthermore, the tergite phenotype appears to perturb cells that are proliferating and migrating, and thus such processes that are not occurring in the *Drosophila* eye could be specifically examined in this system.

In our study, a modifier gene approach was used to examine whether pathways shown to alter repeat RNA-mediated pathology in the eye [14], [20] could also modify the tergite phenotype. Initially we examined whether perturbation of Mbl, a known target of repeat RNA-mediated sequestration and contributor to pathology, contributes to the tergite disruption phenotype. If the phenotype was caused by Mbl sequestration, a further reduction in Mbl would be expected to enhance pathology, however, this was not observed with either CUG or CAG expression phenotypes. Thus, Mbl sequestration does not appear to be a major contributor to the common pathway that leads to similar CUG or CAG-mediated tergite disruption. Interestingly, in both CUG expressing lines, the proportion of progeny showing a milder phenotype was reduced when Mbl levels were reduced, while the proportion with a stronger phenotype was not significantly changed. Pathways involving Mbl sequestration may therefore make a mild, or indirect, contribution to CUG specific pathology, perhaps through the role of Mbl in RNA processing and stability [15], [62]. Structural studies show that each repeat sequence has distinct binding abilities that may account for the CUG specificity in this case [63].

We previously reported that co-expression of complementary CAG and CUG repeat RNA expression transgenes in the eye gives a strong phenotype, that is suppressed by reducing Dcr-2 levels [20]. However, expression of either CAG or CUG repeat RNA transgenes alone, in the absence of a complementary transcript and thus acting as a single-stranded ‘hairpin’ RNA only, gives no eye phenotype [20]. The tergite phenotype therefore enabled us, for the first time, to examine whether single-stranded ‘hairpin’ RNA-mediated cellular perturbation is also suppressed by reducing Dcr-2. In contrast to double-stranded repeat RNA mediated pathology, reduced Dcr-2 levels were not rate limiting for either CUG or CAG-mediated tergite phenotypes [20]. This result suggests that mechanisms responsible for ‘hairpin’ RNA-mediated tergite pathology are not identical to those involved in double-stranded repeat RNA mediate pathology. For technical reasons it was not possible to examine progeny homozygous for the Dcr-2 loss of function allele, and the participation of this enzyme in RNAi processing limits the feasability of reducing Dcr-2 function via this targeted RNAi approach. Thus, we cannot exclude the possibility that some level of Dcr-2 processing, of CUG or CAG ‘hairpin’ RNA, may be necessary for the phenotype. However, our results suggest that even if Dcr-2 is required in both double-stranded and ‘hairpin’-mediated pathology, different dynamics must be involved. Further studies will be necessary to dissect the mechanisms involved in each case.

We further examined whether Dcr-1 processing is rate limiting for the tergite phenotype. Reduced Dcr-1 levels gave opposing effects, with a suppression observed with CUG transgene expression and an enhancement with CAG transgene expression. If pathology occurs through a mechanism dependent on the processing of ‘hairpin’ repeats by Dcr-1, it would be expected that a reduction in processing would give a similar effect in each case. In contrast, the ability to induce opposing effects is suggestive of a more complex interaction. It is unclear how this may occur, however these results highlight the need to further investigate Dcr-1 processing as a candidate pathogenic pathway. Future studies examining other small RNA processing components, such as the Argonaut proteins, may be necessary to understand how this pathway may differentially interact with CUG or CAG repeat-mediated pathology. We have previously reported that double-stranded repeat RNA may cause pathology through the alteration of endogenous miRNA levels [20]. Thus, a possible interaction of ‘hairpin’ RNA with Dcr-1 processing pathways, known to regulate miRNA processing, may provide a candidate pathway that is a common downstream target of ‘hairpin’ and double-stranded RNA-mediated pathology.

Finally, we examined the localisation of ‘hairpin’ repeat RNA in our system, as the formation of nuclear foci has previously been shown to be a hallmark of ‘hairpin’ RNA pathology [61]. Results indicate, that in our model system, CUG expression is sufficient to produce certain, specific RNA foci, while CAG expression is not. Expression of non-hairpin-forming CAA repeat RNA led to identical nuclear staining as with CAG expression. Thus, the ability to form a ‘hairpin’ structure is not sufficient to enable interactions required for the formation of cell specific foci observed with CUG RNA expression. CAG RNA foci have been previously observed in *Drosophila*, mouse and human cells, indicating that CAG expression may induce specific foci under some conditions [15], [18], [42]. These different observations may be due to characteristics of the transgenic expression system. Examining RNA localisation in histoblast cells was not possible for technical reasons, and thus we could not determine whether foci are formed specifically in cells that are perturbed by repeat RNA expression. Nonetheless, CAG transcripts in our system, that did not form specific foci, gave a strong tergite phenotype, and thus are able to undergo as yet undefined interactions necessary to induce pathology. These results, together with our observations of sequence specific modification with Mbl and Dcr-1, are consistent with the possibily that ‘hairpin’ repeat RNA may undergo multiple interactions that contribute to pathology.

The tergite phenotype should be considered in the context of recent findings that non-coding repeat RNA sequences may undergo translation through a repeat-mediated mechanism known as repeat-associated non-ATG (RAN) translation, producing transcripts in all possible reading frames [49]. This process is yet to be observed in *Drosophila*, however, it is possible that homopolymeric peptides are produced at some frequency from our repeat RNA expression constructs including polyglutamine and polyalanine proteins that are known to be pathogenic [8], [49], [50]. Indeed, we previously reported a tergite phenotype due to the expression of a single copy of a transgene encoding a translated GCA repeat, producing a polyalanine peptide [50]. This phenotype may potentially be caused by either the repeat-containing GCA RNA, which is structurally identical to CAG repeat RNA, the presence of polyalanine protein, or a combination of the two. Ubiquitous expression of a polyglutamine encoding CAG repeat is lethal in this system, preventing the analysis of adult tergites [50]. However, expression of a single translated CUG repeat-encoding transgene did not lead to a tergite phenotype [50], perhaps consistent with the weaker CUG phenotypes observed in this current study. As positively confirming the presence of homopolymeric peptides in multiple reading frames is technically complex, further analysis will be required to determine the relative contribution of RNA and homopolymeric protein to the tergite phenotype.

Recent findings suggest that multiple pathways, mediated by ‘hairpin’ and double-stranded repeat RNA, as well as homopolymeric protein tracts, are able to independently cause pathology in *Drosophila* models of expanded repeat disease. Here we characterise a *Drosophila* tergite disruption phenotype caused by CUG or CAG ‘hairpin’ repeat RNA, providing a new, independent system in which to study common pathways that lead to pathology. *Drosophila* models such as this will enable the genetic pathways contributing to each type of repeat-mediated pathology to be defined. Identifying conserved pathways will provide candidates for future studies in vertebrate models, as well as defining ‘molecular hallmarks’ that may be used to verify the contribution of specific pathways modelled in *Drosophila* to human disease.

## Materials and Methods

### Drosophila Stocks

Stocks were obtained from the Bloomington Drosophila Stock Centre (Bloomington, IN, USA) unless otherwise noted. Nomenclature used for *Drosophila* genotypes is as used on Flybase (www.flybase.org). *da-GAL4* (Bloomington stock #8641) was originally described in [54]. *Act5c-GAL4* (Bloomington stock #4414) was originally described in [64]. *T155-GAL4* (Bloomington stock #5076) was originally described by [57]. *Dcr-1^Q1147X^* and *Dcr-2^L811fsX^* were obtained from Professor Richard Carthew and are described in [60]. *mbl^E27^* (Bloomington stock #7318) is caused by imprecise excision of a P-element, removing exon 1 and 2 [27].

### Repeat Expression Lines

Generation of repeat constructs has been previously described [8], [17]. Generation of rCAG_∼100_ and rCUG_∼100_ four transgene copy lines, and nomenclature is described in [20]. Identical methods were used to generate *4xrCAA_∼100_.* To generate GFP-repeat expression lines previously described expanded CAG_∼100_, CTG_∼100_ and CAA_∼100_ constructs were subcloned into pBKS+ vector (GenBank/EMBL accession number X52327) and subsequently inserted into pBD1010 (kindly donated by Professor Barry Dickson) upstream of GFP using EcoRI and XhoI restriction sites. The expanded repeat together with GFP was then cloned into pUAST-attB [65] using EcoRI and XbaI restriction sites. The length of the repeats and the integrity of GFP were confirmed by DNA sequencing.

### Quantification of Tergite Disruption

Newly eclosed adult females were scored for tergite disruption by examining the dorsal abdomen under a standard dissecting microscope. The phenotype was categorised on the scale: 1, like wild-type; 2, tergites mildly disrupted but not split; 3, at least one tergite split; 4, two or more tergites split. Counts from multiple crosses scored under identical conditions were pooled to give a final tally for each genotype. To compare the effect of modifiers on the tergite phenotype categories were pooled into 2 groups, those in category one or two, and those in category three or four. This represents those with a mild, or no phenotype, and those with a strong phenotype, and appeared to be the most robust way to determine if modification was significant. Statistical significance was determined using Fisher’s exact test (GraphPad Prism). This enabled direct comparison of different sized populations, and determined the probability that the distribution of progeny within categories differ between genotypes by chance alone. p = 0.05 was used as a cut off for significance.

Images of the adult abdomen were taken using an Olympus SZX7 microscope fitted with a SZX-AS aperture. Images were captured using an Olympus ColourView IIIU Soft Imaging System camera and AnalysisRuler image acquisition software. Adobe Photoshop CS was used for image preparation.

### *In situ* Hybridisation of Frozen Sections

Whole wandering third instar larvae were frozen in Tissue-Tek® O.C.T.™ freezing medium and a Leica CM1900 cryostat was used to cut 10 mm sections. For each genotype multiple larvae were frozen per mould such that each section contained multiple animals. Prior to hybridisation sections were fixed 15 minutes in ice cold 4% paraformaldehyde in PBS, washed 3×15 minutes in room temperature PBS and quickly rinsed in 100% ethanol. Slides were dried and hybridised for 2 hours, or overnight at 37°C in a humid chamber with 0.5 ng/ul fluorescent oligonucleotide probe in hybridisation buffer (4×SSC, 0.2 g/mL dextran sulphate, 50% formamide, 0.25 mg/mL poly(A) RNA, 0.25mg/mL single stranded DNA, 0.1 M DTT, 0.5× Denhardt’s reagent). Slides were washed 2×15 minutes in 2×SSC, 2×15 minutes in 0.5xSSC at 37°C, air-dried and mounted in Vectashield™ (Vector labs) with 1 ng/mL DAPI to visualise nuclei. Fluorescent microscopy for *in situ* hybridisation experiments was performed using a Zeiss Axioplan 2 microscope with 63× PlanApo objective. Images were captured using Axiovision 4.5 software with an Axiocam MRm camera. Further preparation of images was done using Axiovision, or Adobe Photoshop CS. Multiple nuclei from multiple sections, each containing multiple animals (n≈10) were examined per genotype. Independent transgenic lines were examined for each repeat construct.

### Fluorescent Probes for *in situ* Hybridisation Experiments

Cy3-CTG_10_: Cy3-CTGCTGCTGCTGCTGCTGCTGCTGCTGCTG.

Cy3-CAG_10_: Cy3-CAGCAGCAGCAGCAGCAGCAGCAGCAGCAG.

Cy3-TTG_10_: Cy3-TTGTTGTTGTTGTTGTTGTTGTTGTTGTTG.

Cy3-GFP: Cy3-CCTTCACCCTCTCCACTGACAGAAAATTTGTGCCC.

## Supporting Information

Figure S1 **Graphs show the proportion of progeny within each category for all genotypes.** Proportion (0.0 to 1.0) is shown on the y-axis while each category (1–4) is shown on the x-axis. Total population size, n, is indicated above each graph. **A – K**, phenotype when each line is ubiquitously expressed with da-GAL4. **A’ – H’**, phenotype when the same lines are crossed to *w^1118^* to give progeny with all four repeat transgenes, in the absence of GAL4 driven expression. **A, A’** *w^1118^* wild-type lines. **B, B’** *4xUAS* control line. **C, C’** *4xrCAA_∼100_ [line 1]* and **D, D’** *4xrCAA_∼100_ [line 2].* **E, E’** *4xrCUG_∼100_ [line 1]* and **F, F’** *4xrCUG_∼100_ [line 2].* **G, G’** *4xrCAG_∼100_ [line 1]* and **H, H’** *4xrCAG_∼100_ [line 2]*. **I**, *2xrCAG_∼100_ [line 1A]* and **J,** *2xrCAG_∼100_ [line 1B]*, the two transgene copy lines that were used to create *4xrCAG_∼100_ [line 1].* When each of the two copies is expressed via *da-GAL4*, **I, J**, the resultant phenotype is weaker than in the 4 copy line, **G**.
(TIF)

Figure S2 **Complete data sets showing proportion of progeny within each tergite phenotype category when repeat constructs were expressed in histoblasts with** ***T155–GAL4*** **.** Population size, n, is shown above each graph. Significance indicated is based on comparing each repeat expression line to the EV control, using Fisher’s exact test to compare the distribution of progeny between those with any phenotype (category 2, 3 and 4) and those like wild-type (category 1). *p<0.05 and ***p<0.001. Only *4xrCAG_∼100_* expression in, **F, G**, gives a significant phenotype.
(TIF)

Figure S3 **Effect of reducing Mbl levels on the tergite phenotype.** Each repeat line was expressed ubiquitously via *da-GAL4* and via *da-GAL4* in the presence of one copy of the *mbl^E27^* allele. **A, B, C,** expression of independent lines for each repeat construct, either in a Mbl wild-type background (left column), or in the presence of one copy of the *mbl^E27^* allele (right column). **A**, *w^1118^* control, and two independent *4xrCAA_∼100_* lines, **B**, two independent *4xrCUG_∼100_* lines and **C,** two independent *4xrCAG_∼100_* lines. **D–G**, statistical comparison (Fisher’s exact test) of the proportion of progeny with any phenotype (category 2, 3, 4) and a strong phenotype (category 3, 4) for **D**, *4xrCUG_∼100_ [line 1],* **E**, *4xrCUG_∼100_ [line 2],* **F**, *4xrCAG_∼100_ [line 1]* and **G**, *4xrCAG_∼100_ [line 2].* *p<0.05, **p<0.01, ***p<0.001.
(TIF)

Figure S4 **Effect of reducing Dcr-2 levels on the tergite phenotype.** Each repeat line was expressed ubiquitously via *da-GAL4* and via *da-GAL4* in the presence of one copy of the *dcr2^L811fsX^* allele. **A, B, C**, expression of independent lines for each repeat construct, either in a Mbl wild-type background (left column), or in the presence of one copy of the *mbl^E27^* allele (right column). A, *w^1118^* control, and two independent *4xrCAA_∼100_* lines, **B**, two independent *4xrCUG_∼100_* lines and **C**, two independent *4xrCAG_∼100_* lines. **D–G**, statistical comparison (Fisher’s exact test) of the proportion of progeny with any phenotype (category 2, 3, 4) and a strong phenotype (category 3, 4) for **D**, *4xrCUG_∼100_ [line 1],* **E**, *4xrCUG_∼100_ [line 2],* **F**, *4xrCAG_∼100_ [line 1]* and **G**, *4xrCAG_∼100_ [line 2].* *p<0.05, **p<0.01, ***p<0.001.
(TIF)

Figure S5 **Effect of reducing Dcr-1 levels on the tergite phenotype.** Each repeat line was expressed ubiquitously via *da-GAL4* and via *da-GAL4* in the presence of one copy of the *dcr1^Q1147X^* allele. **A, B, C**, expression of independent lines for each repeat construct, either in a Mbl wild-type background (left column), or in the presence of one copy of the *mbl^E27^* allele (right column). **A**, *w^1118^* control, and two independent *4xrCAA_∼100_* lines, **B,** two independent *4xrCUG_∼100_* lines and **C**, two independent *4xrCAG_∼100_* lines. **D–G**, statistical comparison (Fisher’s exact test) of the proportion of progeny with any phenotype (category 2, 3, 4) and a strong phenotype (category 3, 4) for **D**, *4xrCUG_∼100_ [line 1],* **E**, *4xrCUG_∼100_ [line 2]*, **F**, *4xrCAG_∼100_ [line 1]* and **G**, *4xrCAG_∼100_ [line 2].* *p<0.05, **p<0.01, ***p<0.001.
(TIF)

Figure S6 **Cellular localization of the** ***rCUG_∼100_*** **transcript.** **A,** Schematic of the *rCUG_∼100_* transcript (not to scale). A short non-functional peptide (black) is encoded upstream of the repeat (blue) which is within the 3′UTR (dotted line). Probes were designed to be complementary to the repeat, in this case a Cy3-CAG10 probe targets the CUG_∼100_ repeat. **B-F**, Microscope images (63x) of larval muscle cells probed with the Cy3-CAG_10_ probe. Left panel shows the Cy3 signal alone, right panel shows a merge of the Cy3 signal (red) and DAPI (blue) to label nuclei. **B**, *da-GAL4*/+ larvae show no Cy3 signal. **C**, +/*4xrCUG_∼100_ [line 1]* progeny with four transgenes but no GAL4 driver show no Cy3 signal. **D**, *da-GAL4* driven expression of *4xrCUG_∼100_ [line 1]* leads to many foci throughout the nucleus. **E**, +/*4xrCUG_∼100_ [line 2]* progeny with no GAL4 driven expression show no signal, while, **F**, expression of *4xrCUG_∼100_ [line 2]* via *da-GAL4* leads to multiple nuclear foci.
(TIF)

Figure S7 **Cellular localization of the** ***rCAG_∼100_*** **transcript.** **A**, Schematic of the *rCAG_∼100_* transcript (not to scale). A short non-functional peptide (black) is encoded upstream of the repeat (blue) which is within the 3′UTR (dotted line). Probes were designed to be complementary to the repeat, in this case a Cy3-CTG_10_ probe targets the *rCAG_∼100_* repeat. **B–F**, Microscope images (63x) of larval muscle cells probed with the Cy3-CTG_10_ probe. Left panel shows the Cy3 signal alone, right panel shows a merge of the Cy3 signal (red) and DAPI (blue) to label nuclei. **B**, *da-GAL4*/+ larvae show a weak Cy3 signal due to background staining (asterisk). **C**, +/*4xrCAG_∼100_ [line 1]* progeny with four transgenes but no GAL4 driver show only weak background staining. **D**, *da-GAL4* driven expression of *4xrCAG_∼100_ [line 1]* leads to only one to four foci (arrowheads) throughout the nucleus. **E**, +/*4xrCAG_∼100_ [line 2]* progeny with no GAL4 driven expression show only weak background staining, while, F, expression of *4xrCAG_∼100_ [line 2]* via *da-GAL4* leads to only a small number of foci (arrowheads).
(TIF)

Figure S8 **Cellular localization of the** ***rCAA_∼100_*** **transcript. A**, Schematic of the *rCAA_∼100_* transcript (not to scale). A short non-functional peptide (black) is encoded upstream of the repeat (blue) which is within the 3′UTR (dotted line). Probes were designed to be complementary to the repeat, in this case a Cy3-TTG10 probe targets the CAA∼100 repeat. **B-F**, Microscope images (63x) of larval muscle cells probed with the Cy3-TTG10 probe. Left panel shows the Cy3 signal alone, right panel shows a merge of the Cy3 signal (red) and DAPI (blue) to label nuclei. **B**, *da-GAL4*/+ larvae show only weak background staining. **C**, +/*4xrCAA_∼100_ [line 1]* progeny carrying four transgenes but no GAL4 driver show only weak background staining (asterisk). **D**, *da-GAL4* driven expression of *4xrCAA_∼100_ [line 1]* leads to one to four foci (arrowheads) throughout the nucleus. **E**, +/*4xrCAA_∼100_ [line 2]* progeny with no GAL4 driven expression show only weak background staining, while, **F**, expression of *4xrCAA_∼100_ [line 2]* via *da-GAL4* leads to one to four foci (arrowheads).
(TIF)

Table S1 **Viability when each repeat is expressed via** ***da-GAL4*** **A at 25°C and B at 29°C.** For each genotype total population size (n) is shown along with number of progeny that express four copies of the transgene, and number that inherit the compound balancer chromosome. Proportion with four copies of the transgene, and 95% confidence interval (based on a binomial distribution) for the particular proportion are shown. P values are given for Fisher’s exact test using the raw values comparing the number of 4x transgene, and balancer progeny for each genotype to either the *4xUAS* control, or *4xrCAA_∼100_ [line 1].*
(TIF)

Table S2 **Tergite phenotypes with the ubiquitous** ***Act5c-GAL4*** **driver.** Analysis of tergite phenotypes when repeat lines are driven with the ubiquitous *Act5c-GAL4* driver. Phenotype strength is based on a qualitative scale (mild, moderate, severe) where severe represents the worst phenotype observed of all lines, and cannot be compared directly to *da-GAL4* quantitative results. Relative severities appear to be approximately comparable between drivers where *rCAG_∼100_ [line 1]* gave the most severe tergite phenotype with both *da-GAL4* and *Act5c-GAL4*. Similarly, as for *da-GAL4, rCAG_∼100_ [line 1]* and *rCUG_∼100_ [line 2]* showed reduced viability.
(TIF)

Table S3 **Distribution of progeny between phenotype categories in all repeat lines with and without** ***da-GAL4 driver*** **.** Proportion of total progeny (n) for each genotype that fall within each phenotype scoring category.
(TIF)

Table S4 **Effect on distribution of progeny between categories with and without** ***mbl^E27^*** **.** Each repeat line was expressed ubiquitously via *da-GAL4* and via *da-GAL4* in the presence of one copy of the *mbl^E27^* allele. Table shows the total number of flies scored for each genotype (n), and the proportion of the total represented by each phenotype category where 0.000 is no progeny in that category and 1.000 is all progeny in that category.
(TIF)

Table S5 **Effect on distribution of progeny between categories with and without** ***Dcr-2^L811fsX^*** **.** Each repeat line was expressed ubiquitously via *da-GAL4* and via *da-GAL4* in the presence of one copy of the *Dcr-2^L811fsX^* allele. Table shows the total number of flies scored for each genotype (n), and the proportion of the total represented by each phenotype category where 0.000 is no progeny in that category and 1.000 is all progeny in that category.
(TIF)

Table S6 **Effect on distribution of progeny between categories with and without** ***Dcr-1^Q1147X^*** **.** Each repeat line was expressed ubiquitously via *da-GAL4* and via *da-GAL4* in the presence of one copy of the *Dcr-1^Q1147X^* allele. Table shows the total number of flies scored for each genotype (n), and the proportion of the total represented by each phenotype category where 0.000 is no progeny in that category and 1.000 is all progeny in that category.
(TIF)

Table S7 **Statistical comparison of tergite severity when different mutations are introduced.** Tables shows p values from Fisher’s exact test comparing genotypes for the distribution between progeny with any phenotype (category 2, 3 and 4) and others, or between progeny with a strong phenotype (category 3 and 4) and others. In each case comparisons are made to the population expressing each repeat with da-GAL4 alone.
(TIF)
