# Supplementary figures and images for: Ubiquitous Expression of CUG or CAG Trinucleotide Repeat RNA Causes Common Morphological Defects in a Drosophila Model of RNA-Mediated Pathology
Source: PLoS One. 2012 Jun 8;7(6):e38516. doi: 10.1371/journal.pone.0038516 (PMC3371033; doi:10.1371/journal.pone.0038516)

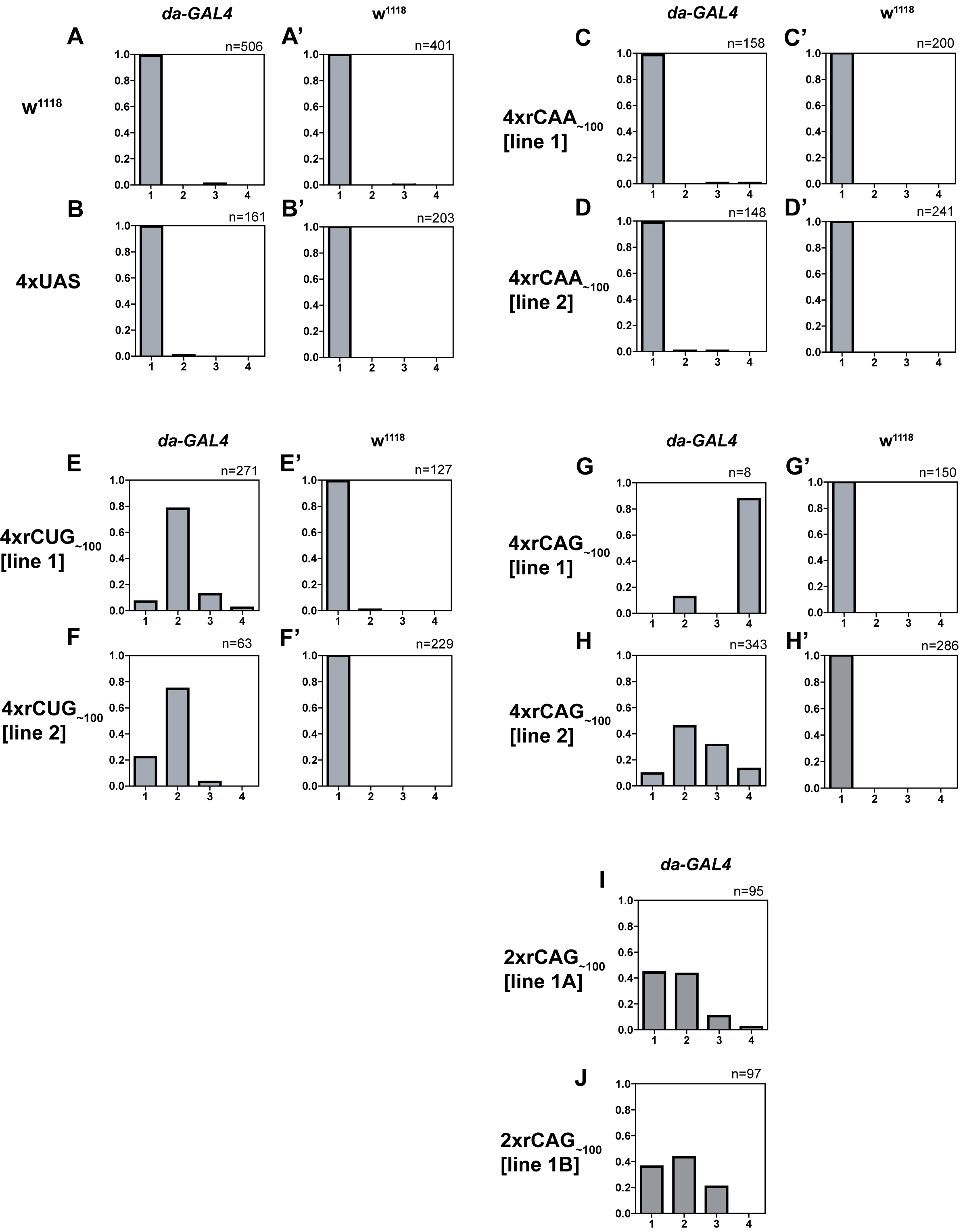

Supplement: Figure S1 — Graphs show the proportion of progeny within each category for all genotypes. Proportion (0.0 to 1.0) is shown on the y-axis while each category (1–4) is shown on the x-axis. Total population size, n, is indicated above each graph. A – K, phenotype when each line is ubiquitously expressed with da-GAL4. A’ – H’, phenotype when the same lines are crossed to w1118 to give progeny with all four repeat transgenes, in the absence of GAL4 driven expression. A, A’ w1118 wild-type lines. B, B’ 4xUAS control line. C, C’ 4xrCAA∼100 [line 1] and D, D’ 4xrCAA∼100 [line 2]. E, E’ 4xrCUG∼100 [line 1] and F, F’ 4xrCUG∼100 [line 2]. G, G’ 4xrCAG∼100 [line 1] and H, H’ 4xrCAG∼100 [line 2]. I, 2xrCAG∼100 [line 1A] and J, 2xrCAG∼100 [line 1B], the two transgene copy lines that were used to create 4xrCAG∼100 [line 1]. When each of the two copies is expressed via da-GAL4, I, J, the resultant phenotype is weaker than in the 4 copy line, G. (TIF) [file pone.0038516.s001.tif]

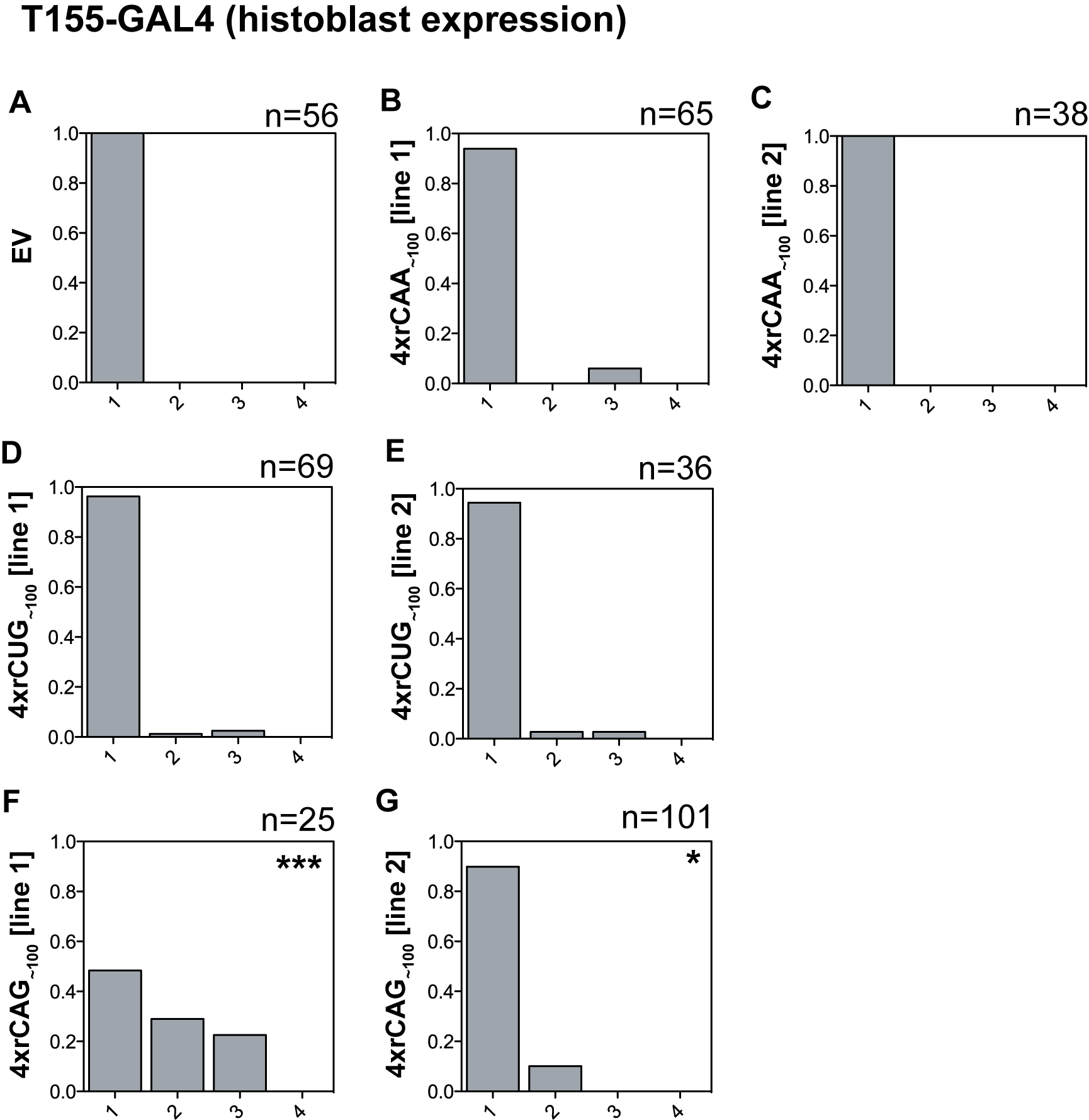

Supplement: Figure S2 — Complete data sets showing proportion of progeny within each tergite phenotype category when repeat constructs were expressed in histoblasts with T155–GAL4 . Population size, n, is shown above each graph. Significance indicated is based on comparing each repeat expression line to the EV control, using Fisher’s exact test to compare the distribution of progeny between those with any phenotype (category 2, 3 and 4) and those like wild-type (category 1). *p<0.05 and ***p<0.001. Only 4xrCAG∼100 expression in, F, G, gives a significant phenotype. (TIF) [file pone.0038516.s002.tif]

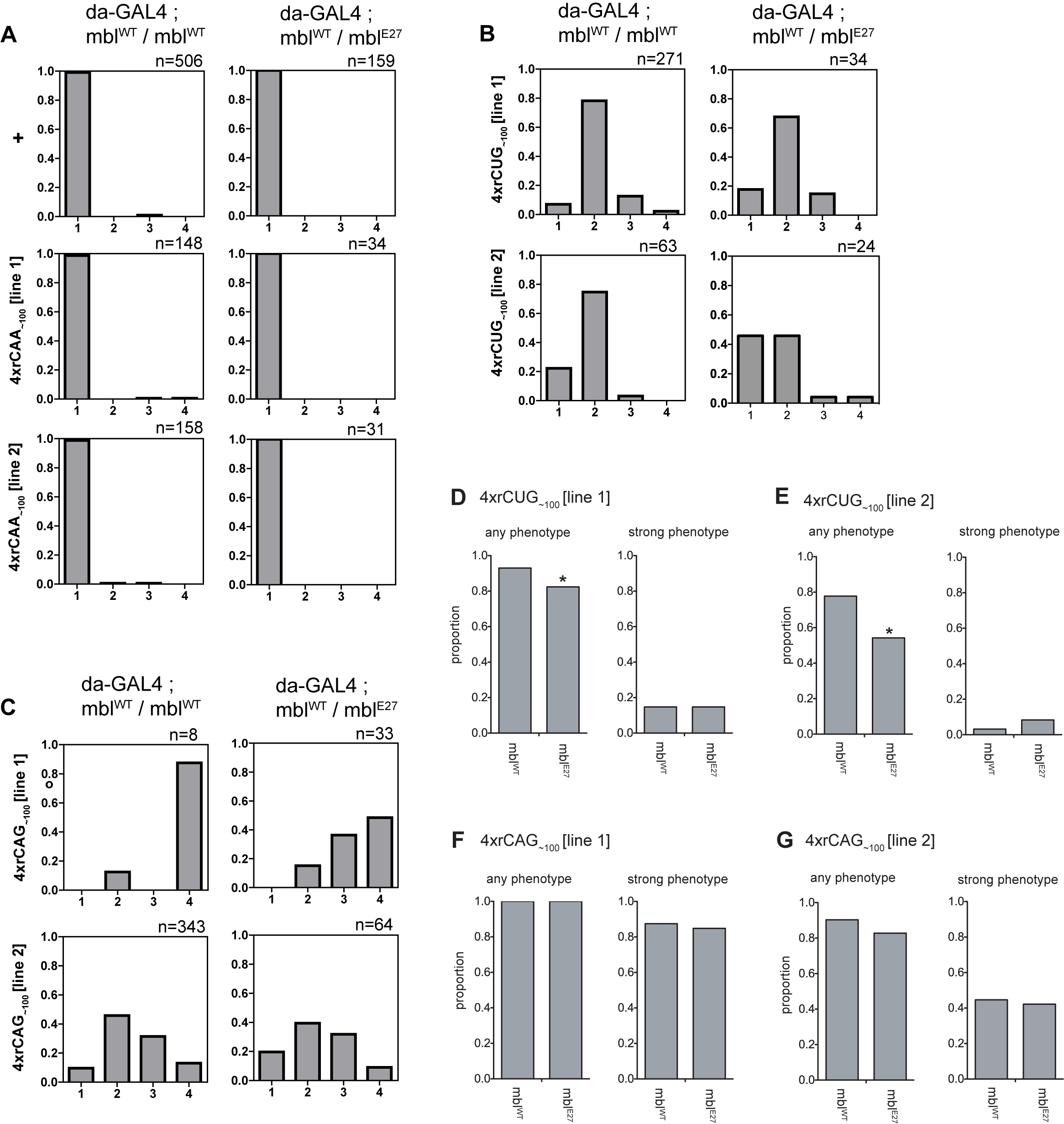

Supplement: Figure S3 — Effect of reducing Mbl levels on the tergite phenotype. Each repeat line was expressed ubiquitously via da-GAL4 and via da-GAL4 in the presence of one copy of the mblE27 allele. A, B, C, expression of independent lines for each repeat construct, either in a Mbl wild-type background (left column), or in the presence of one copy of the mblE27 allele (right column). A, w1118 control, and two independent 4xrCAA∼100 lines, B, two independent 4xrCUG∼100 lines and C, two independent 4xrCAG∼100 lines. D–G, statistical comparison (Fisher’s exact test) of the proportion of progeny with any phenotype (category 2, 3, 4) and a strong phenotype (category 3, 4) for D, 4xrCUG∼100 [line 1], E, 4xrCUG∼100 [line 2], F, 4xrCAG∼100 [line 1] and G, 4xrCAG∼100 [line 2]. *p<0.05, **p<0.01, ***p<0.001. (TIF) [file pone.0038516.s003.tif]

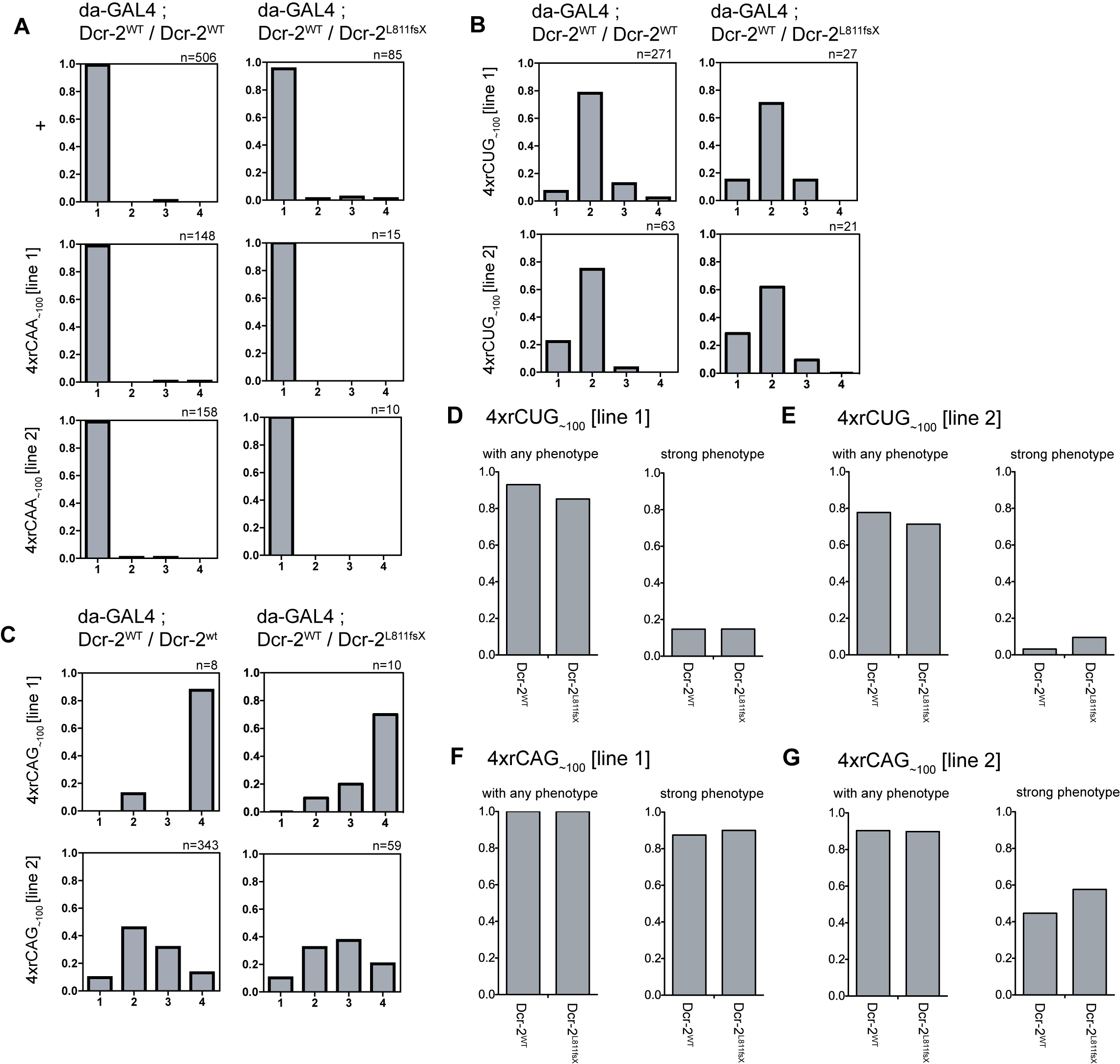

Supplement: Figure S4 — Effect of reducing Dcr-2 levels on the tergite phenotype. Each repeat line was expressed ubiquitously via da-GAL4 and via da-GAL4 in the presence of one copy of the dcr2L811fsX allele. A, B, C, expression of independent lines for each repeat construct, either in a Mbl wild-type background (left column), or in the presence of one copy of the mblE27 allele (right column). A, w1118 control, and two independent 4xrCAA∼100 lines, B, two independent 4xrCUG∼100 lines and C, two independent 4xrCAG∼100 lines. D–G, statistical comparison (Fisher’s exact test) of the proportion of progeny with any phenotype (category 2, 3, 4) and a strong phenotype (category 3, 4) for D, 4xrCUG∼100 [line 1], E, 4xrCUG∼100 [line 2], F, 4xrCAG∼100 [line 1] and G, 4xrCAG∼100 [line 2]. *p<0.05, **p<0.01, ***p<0.001. (TIF) [file pone.0038516.s004.tif]

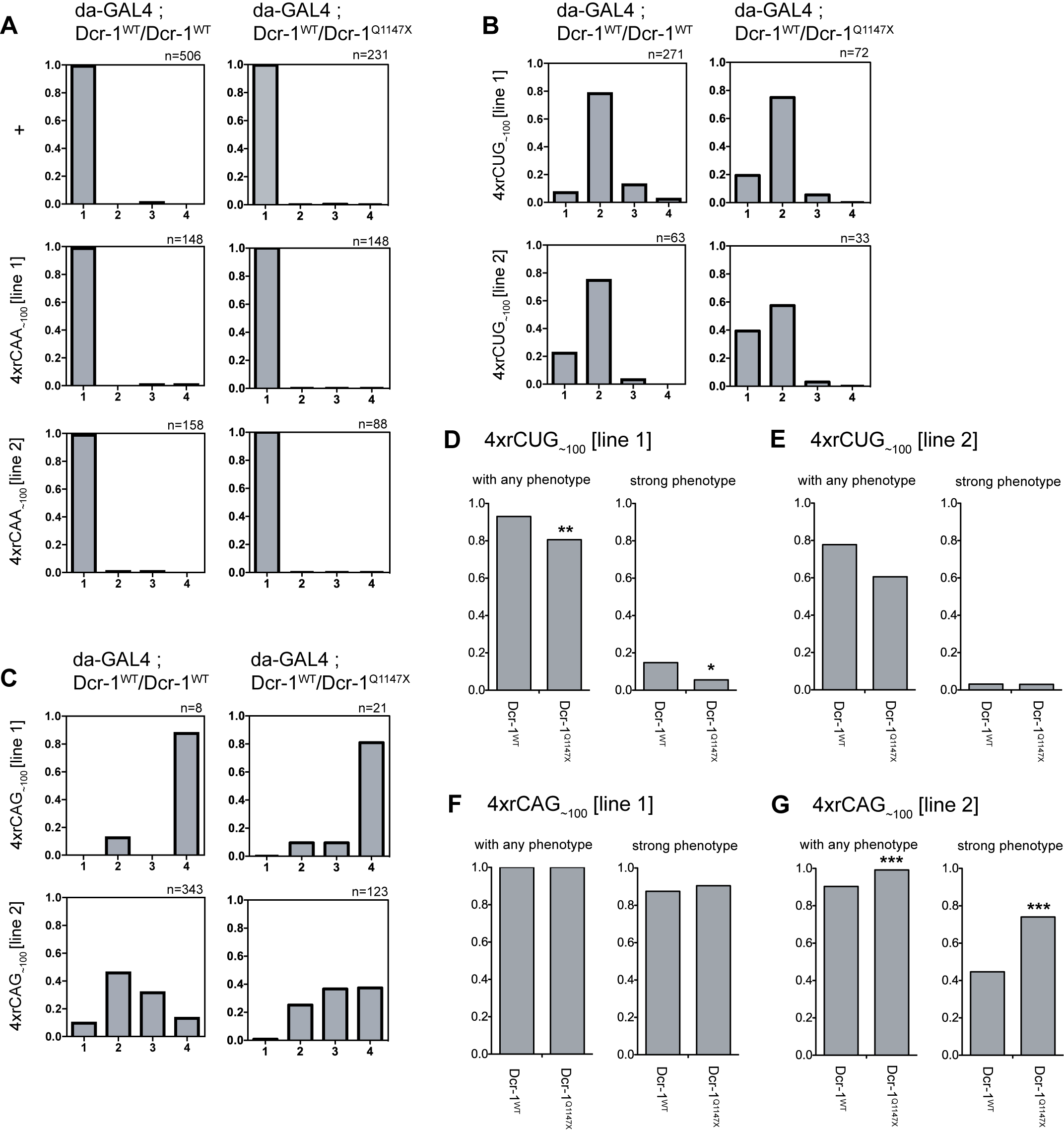

Supplement: Figure S5 — Effect of reducing Dcr-1 levels on the tergite phenotype. Each repeat line was expressed ubiquitously via da-GAL4 and via da-GAL4 in the presence of one copy of the dcr1Q1147X allele. A, B, C, expression of independent lines for each repeat construct, either in a Mbl wild-type background (left column), or in the presence of one copy of the mblE27 allele (right column). A, w1118 control, and two independent 4xrCAA∼100 lines, B, two independent 4xrCUG∼100 lines and C, two independent 4xrCAG∼100 lines. D–G, statistical comparison (Fisher’s exact test) of the proportion of progeny with any phenotype (category 2, 3, 4) and a strong phenotype (category 3, 4) for D, 4xrCUG∼100 [line 1], E, 4xrCUG∼100 [line 2], F, 4xrCAG∼100 [line 1] and G, 4xrCAG∼100 [line 2]. *p<0.05, **p<0.01, ***p<0.001. (TIF) [file pone.0038516.s005.tif]

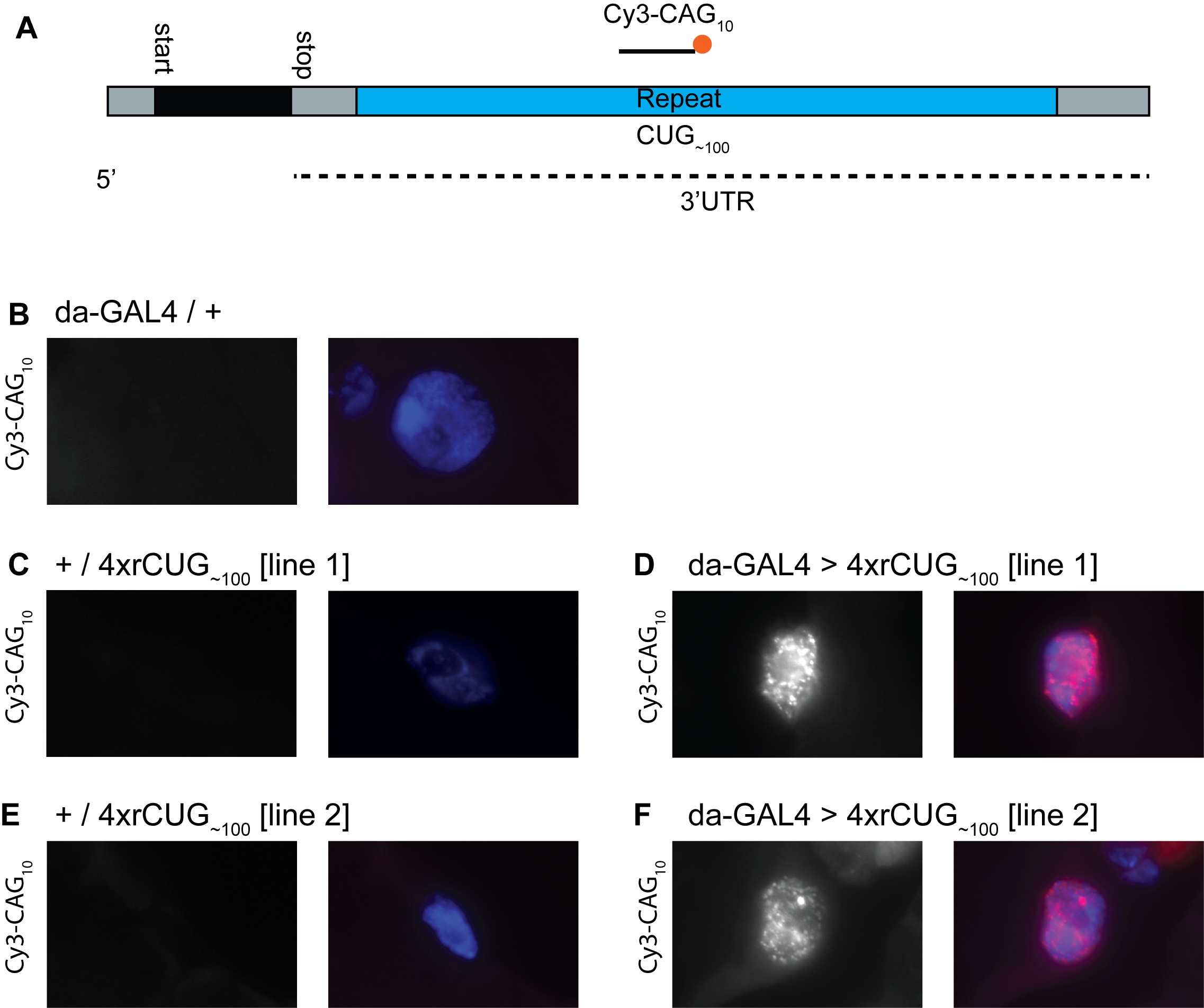

Supplement: Figure S6 — Cellular localization of the rCUG∼100 transcript. A, Schematic of the rCUG∼100 transcript (not to scale). A short non-functional peptide (black) is encoded upstream of the repeat (blue) which is within the 3′UTR (dotted line). Probes were designed to be complementary to the repeat, in this case a Cy3-CAG10 probe targets the CUG∼100 repeat. B-F, Microscope images (63x) of larval muscle cells probed with the Cy3-CAG10 probe. Left panel shows the Cy3 signal alone, right panel shows a merge of the Cy3 signal (red) and DAPI (blue) to label nuclei. B, da-GAL4/+ larvae show no Cy3 signal. C, +/4xrCUG∼100 [line 1] progeny with four transgenes but no GAL4 driver show no Cy3 signal. D, da-GAL4 driven expression of 4xrCUG∼100 [line 1] leads to many foci throughout the nucleus. E, +/4xrCUG∼100 [line 2] progeny with no GAL4 driven expression show no signal, while, F, expression of 4xrCUG∼100 [line 2] via da-GAL4 leads to multiple nuclear foci. (TIF) [file pone.0038516.s006.tif]

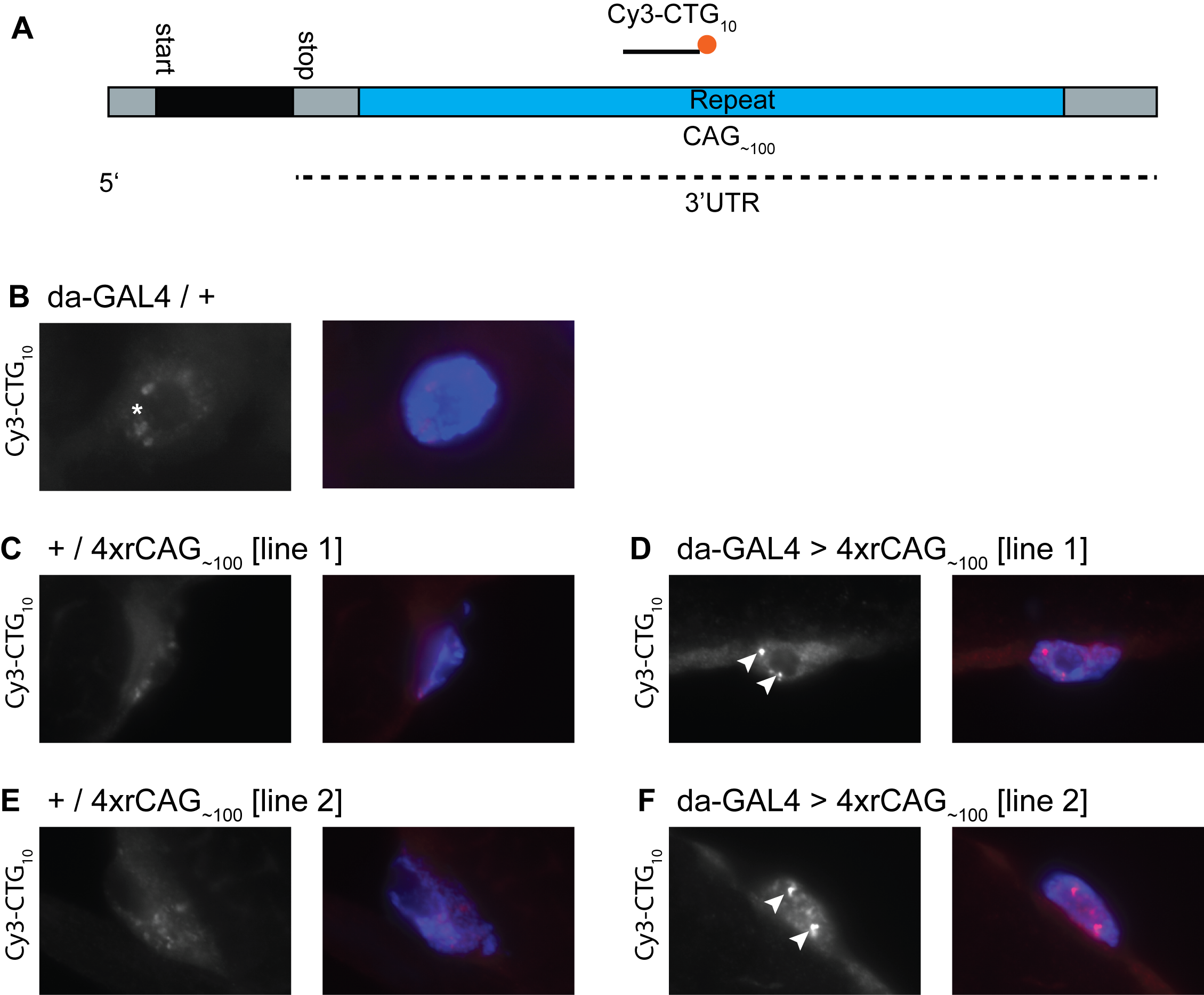

Supplement: Figure S7 — Cellular localization of the rCAG∼100 transcript. A, Schematic of the rCAG∼100 transcript (not to scale). A short non-functional peptide (black) is encoded upstream of the repeat (blue) which is within the 3′UTR (dotted line). Probes were designed to be complementary to the repeat, in this case a Cy3-CTG10 probe targets the rCAG∼100 repeat. B–F, Microscope images (63x) of larval muscle cells probed with the Cy3-CTG10 probe. Left panel shows the Cy3 signal alone, right panel shows a merge of the Cy3 signal (red) and DAPI (blue) to label nuclei. B, da-GAL4/+ larvae show a weak Cy3 signal due to background staining (asterisk). C, +/4xrCAG∼100 [line 1] progeny with four transgenes but no GAL4 driver show only weak background staining. D, da-GAL4 driven expression of 4xrCAG∼100 [line 1] leads to only one to four foci (arrowheads) throughout the nucleus. E, +/4xrCAG∼100 [line 2] progeny with no GAL4 driven expression show only weak background staining, while, F, expression of 4xrCAG∼100 [line 2] via da-GAL4 leads to only a small number of foci (arrowheads). (TIF) [file pone.0038516.s007.tif]

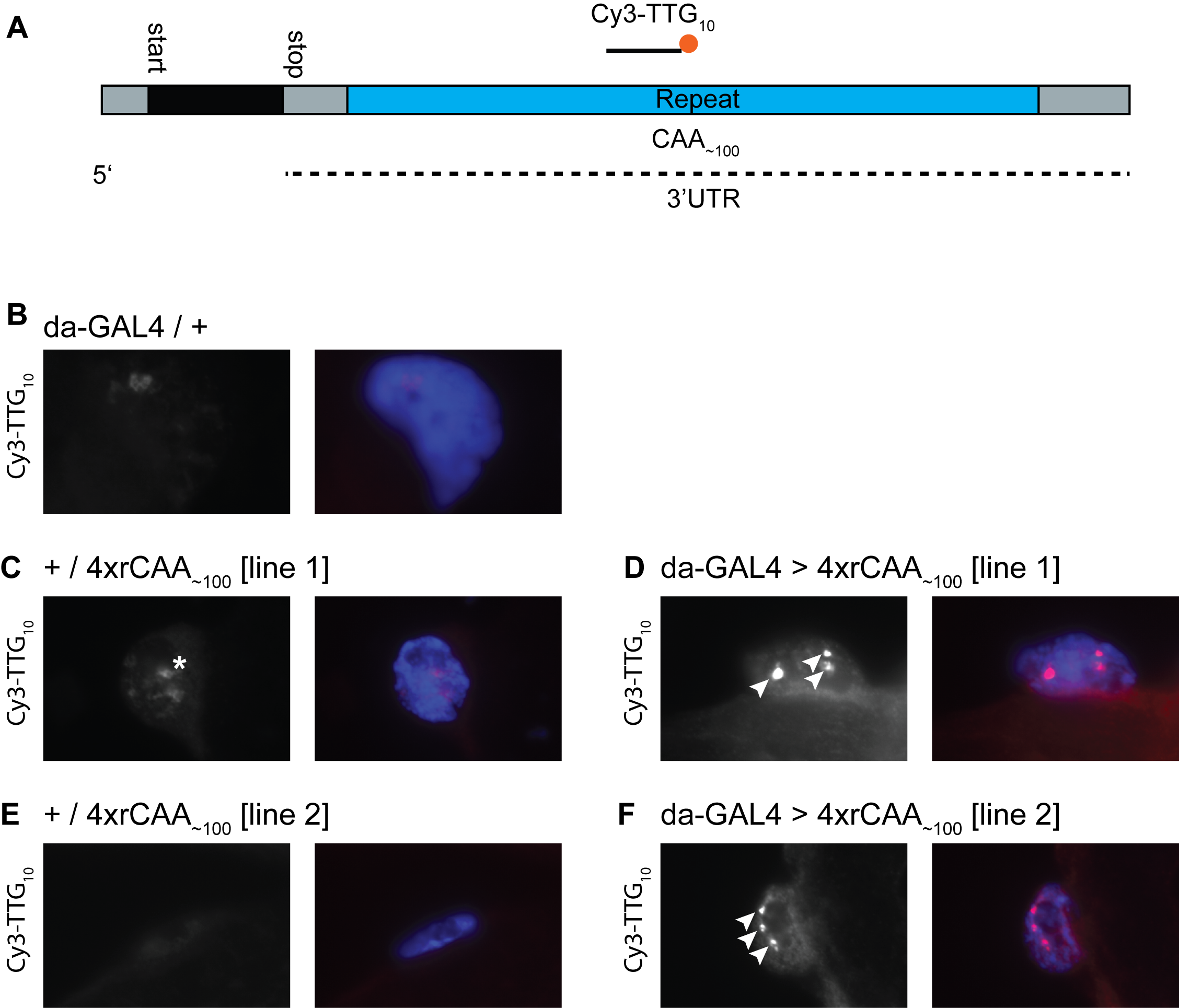

Supplement: Figure S8 — Cellular localization of the rCAA∼100 transcript. A, Schematic of the rCAA∼100 transcript (not to scale). A short non-functional peptide (black) is encoded upstream of the repeat (blue) which is within the 3′UTR (dotted line). Probes were designed to be complementary to the repeat, in this case a Cy3-TTG10 probe targets the CAA∼100 repeat. B-F, Microscope images (63x) of larval muscle cells probed with the Cy3-TTG10 probe. Left panel shows the Cy3 signal alone, right panel shows a merge of the Cy3 signal (red) and DAPI (blue) to label nuclei. B, da-GAL4/+ larvae show only weak background staining. C, +/4xrCAA∼100 [line 1] progeny carrying four transgenes but no GAL4 driver show only weak background staining (asterisk). D, da-GAL4 driven expression of 4xrCAA∼100 [line 1] leads to one to four foci (arrowheads) throughout the nucleus. E, +/4xrCAA∼100 [line 2] progeny with no GAL4 driven expression show only weak background staining, while, F, expression of 4xrCAA∼100 [line 2] via da-GAL4 leads to one to four foci (arrowheads). (TIF) [file pone.0038516.s008.tif]

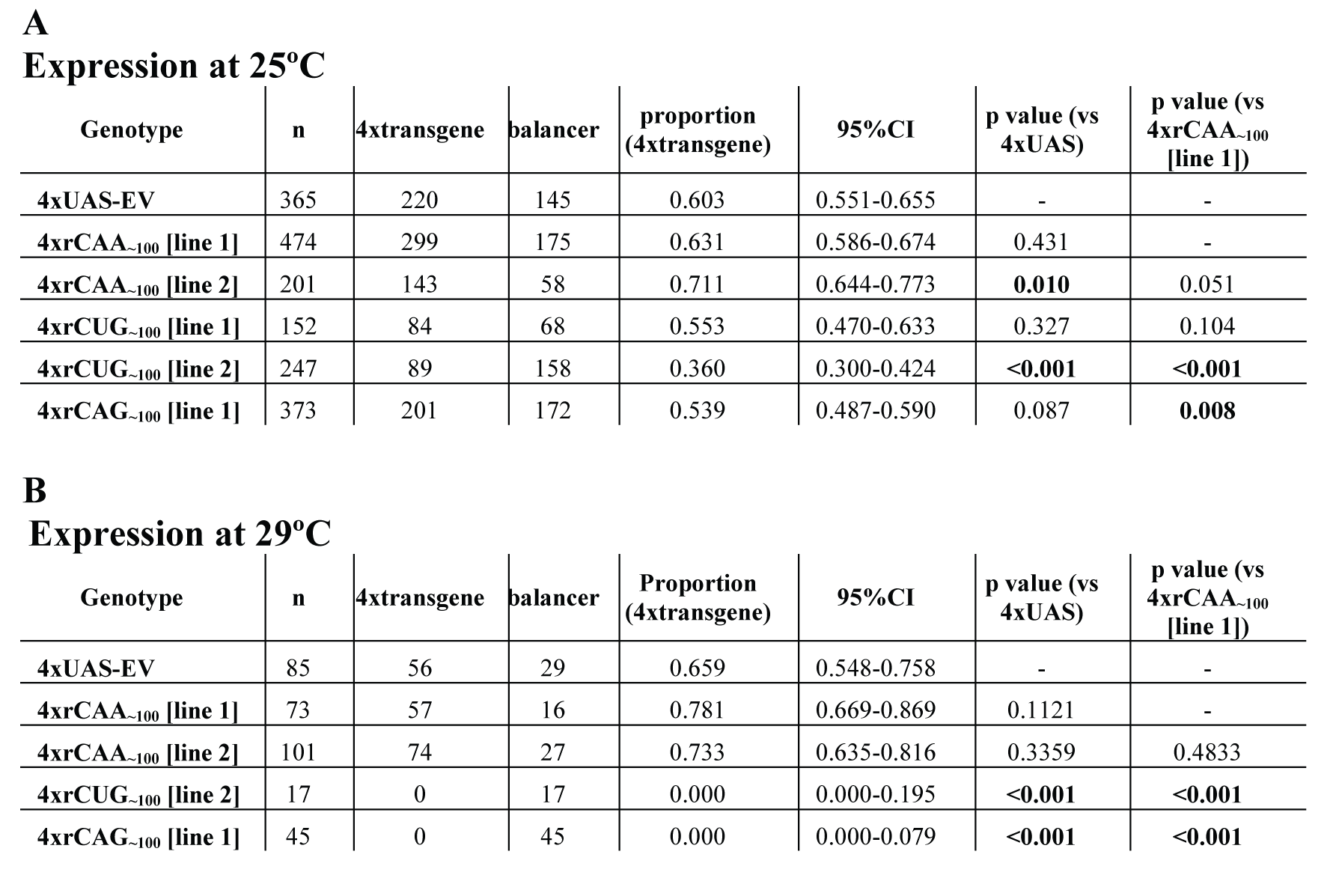

Supplement: Table S1 — Viability when each repeat is expressed via da-GAL4 A at 25°C and B at 29°C. For each genotype total population size (n) is shown along with number of progeny that express four copies of the transgene, and number that inherit the compound balancer chromosome. Proportion with four copies of the transgene, and 95% confidence interval (based on a binomial distribution) for the particular proportion are shown. P values are given for Fisher’s exact test using the raw values comparing the number of 4x transgene, and balancer progeny for each genotype to either the 4xUAS control, or 4xrCAA∼100 [line 1]. (TIF) [file pone.0038516.s009.tif]

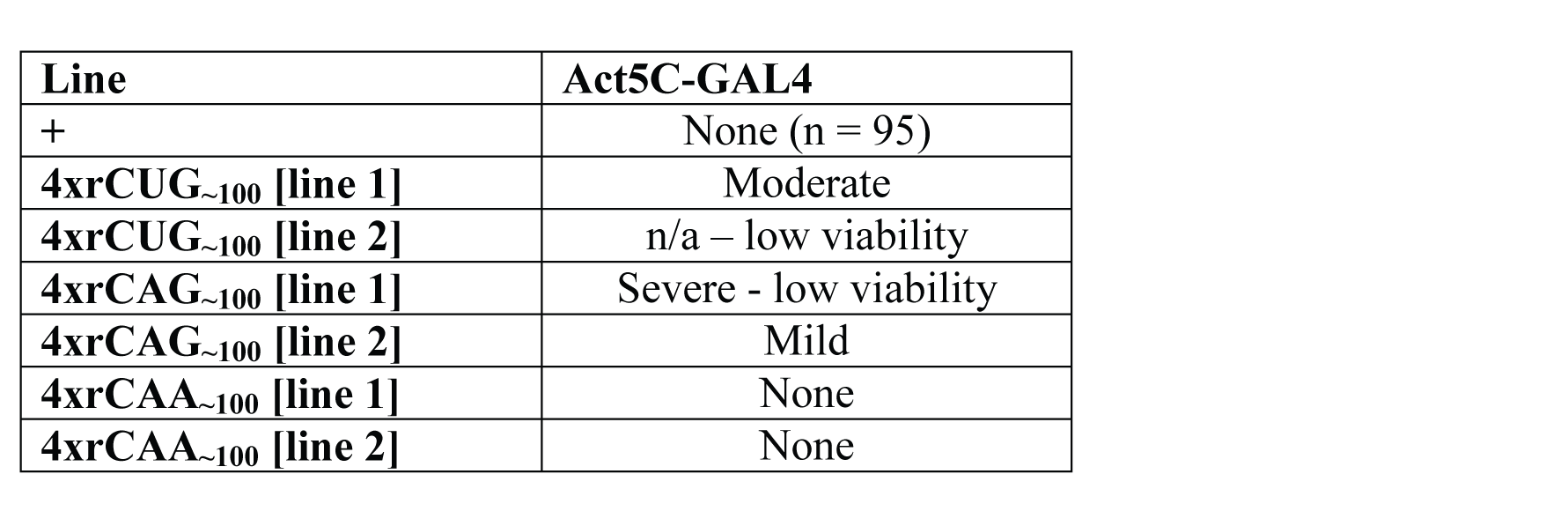

Supplement: Table S2 — Tergite phenotypes with the ubiquitous Act5c-GAL4 driver. Analysis of tergite phenotypes when repeat lines are driven with the ubiquitous Act5c-GAL4 driver. Phenotype strength is based on a qualitative scale (mild, moderate, severe) where severe represents the worst phenotype observed of all lines, and cannot be compared directly to da-GAL4 quantitative results. Relative severities appear to be approximately comparable between drivers where rCAG∼100 [line 1] gave the most severe tergite phenotype with both da-GAL4 and Act5c-GAL4. Similarly, as for da-GAL4, rCAG∼100 [line 1] and rCUG∼100 [line 2] showed reduced viability. (TIF) [file pone.0038516.s010.tif]

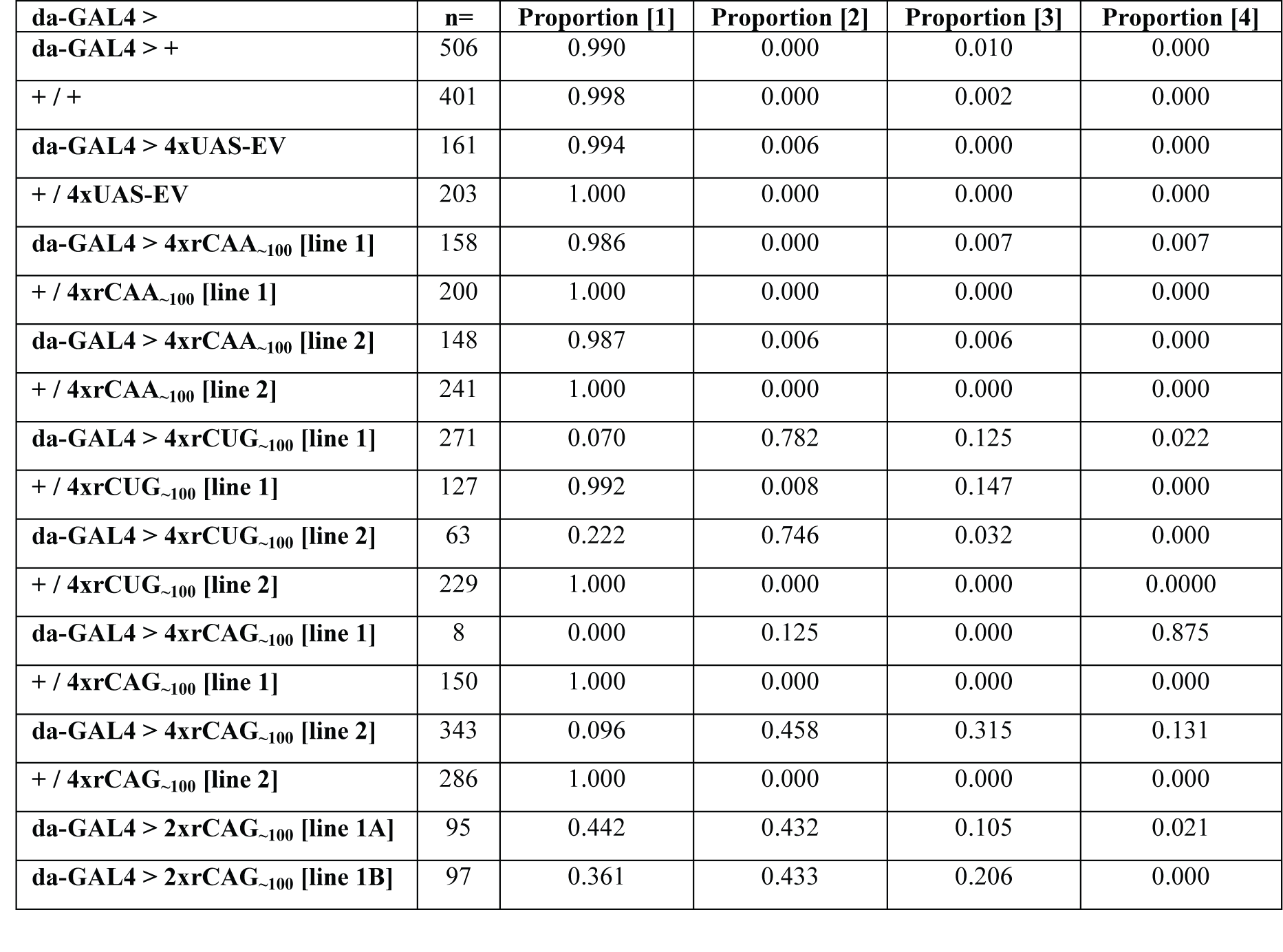

Supplement: Table S3 — Distribution of progeny between phenotype categories in all repeat lines with and without da-GAL4 driver . Proportion of total progeny (n) for each genotype that fall within each phenotype scoring category. (TIF) [file pone.0038516.s011.tif]

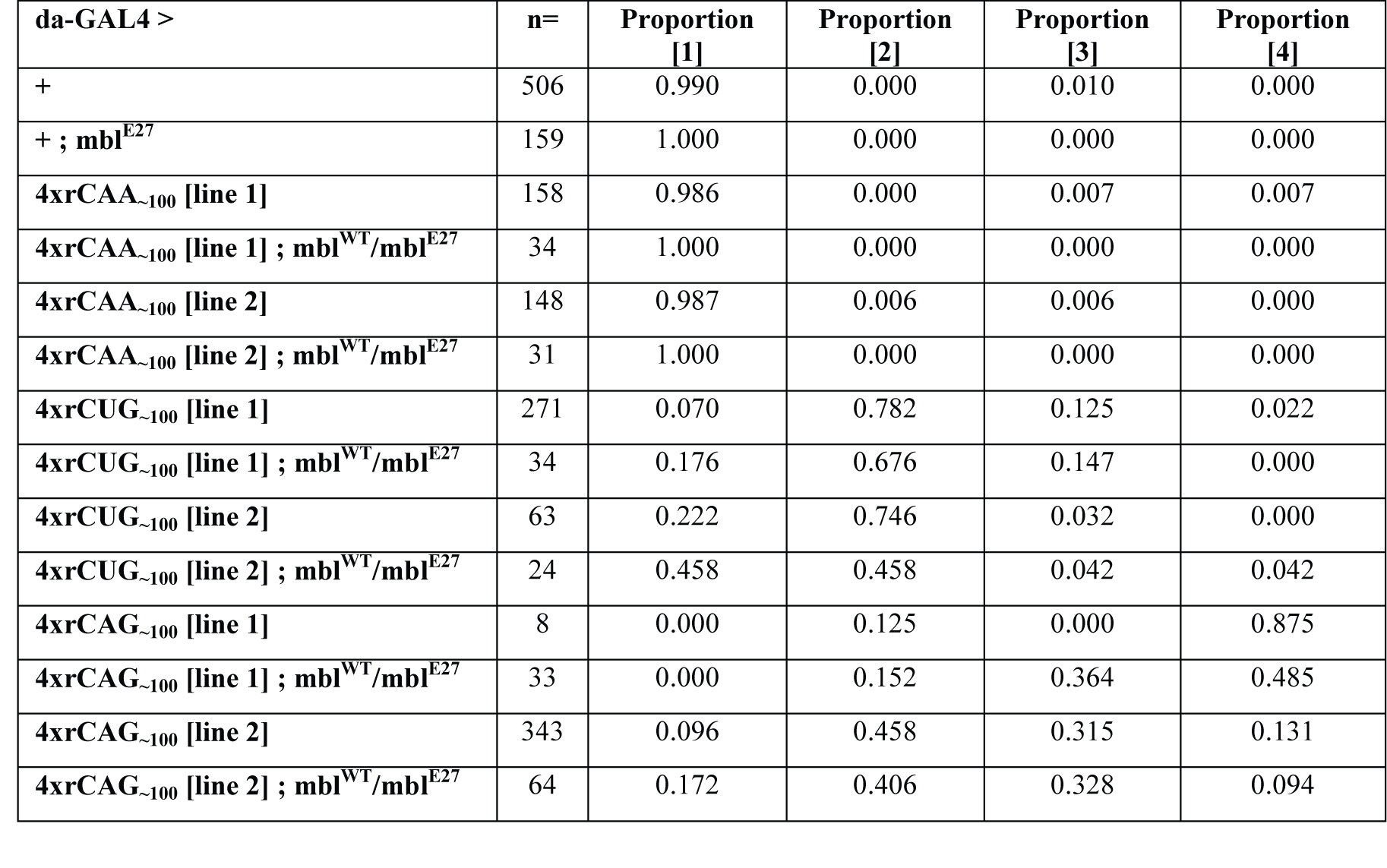

Supplement: Table S4 — Effect on distribution of progeny between categories with and without mblE27 . Each repeat line was expressed ubiquitously via da-GAL4 and via da-GAL4 in the presence of one copy of the mblE27 allele. Table shows the total number of flies scored for each genotype (n), and the proportion of the total represented by each phenotype category where 0.000 is no progeny in that category and 1.000 is all progeny in that category. (TIF) [file pone.0038516.s012.tif]

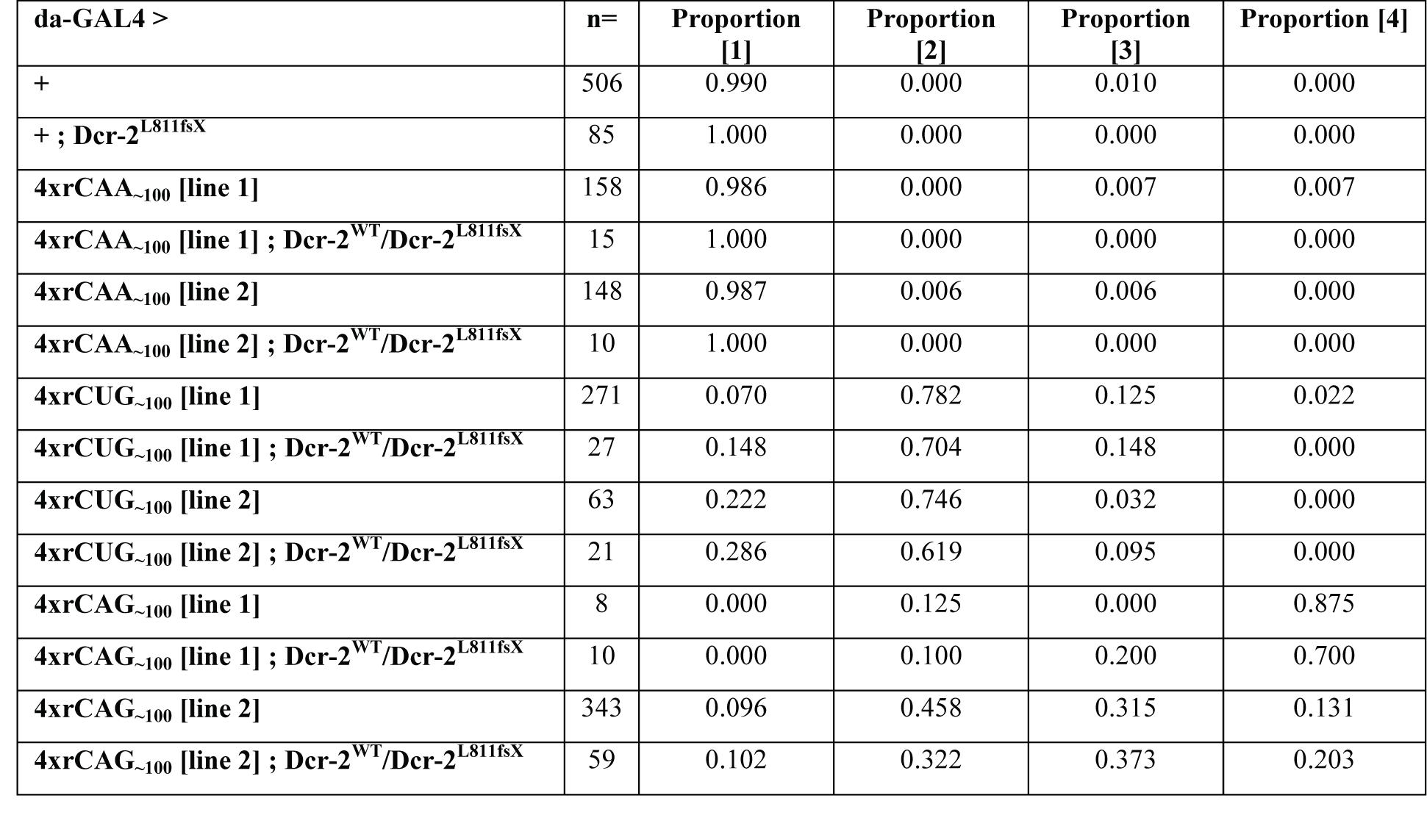

Supplement: Table S5 — Effect on distribution of progeny between categories with and without Dcr-2L811fsX . Each repeat line was expressed ubiquitously via da-GAL4 and via da-GAL4 in the presence of one copy of the Dcr-2L811fsX allele. Table shows the total number of flies scored for each genotype (n), and the proportion of the total represented by each phenotype category where 0.000 is no progeny in that category and 1.000 is all progeny in that category. (TIF) [file pone.0038516.s013.tif]

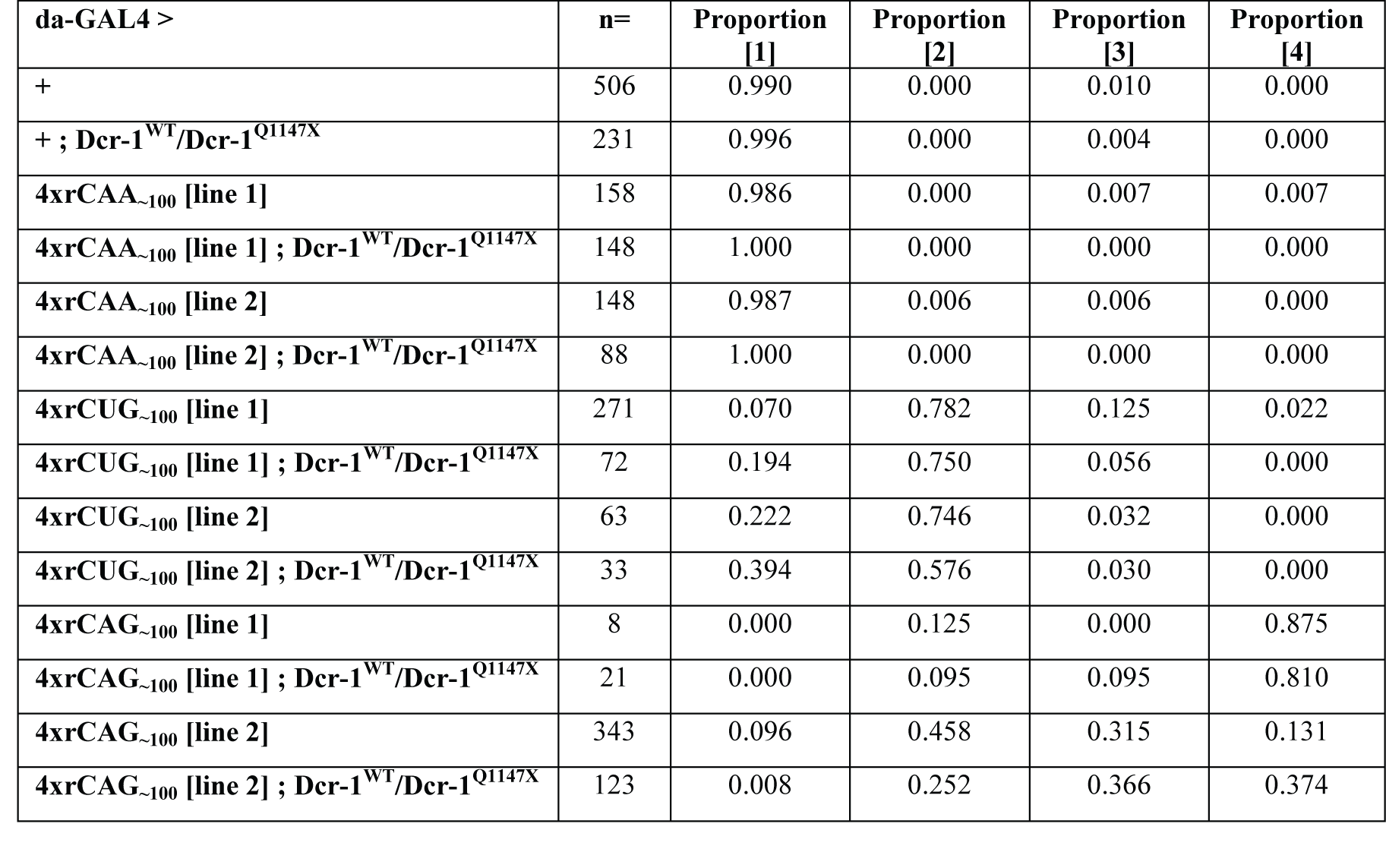

Supplement: Table S6 — Effect on distribution of progeny between categories with and without Dcr-1Q1147X . Each repeat line was expressed ubiquitously via da-GAL4 and via da-GAL4 in the presence of one copy of the Dcr-1Q1147X allele. Table shows the total number of flies scored for each genotype (n), and the proportion of the total represented by each phenotype category where 0.000 is no progeny in that category and 1.000 is all progeny in that category. (TIF) [file pone.0038516.s014.tif]

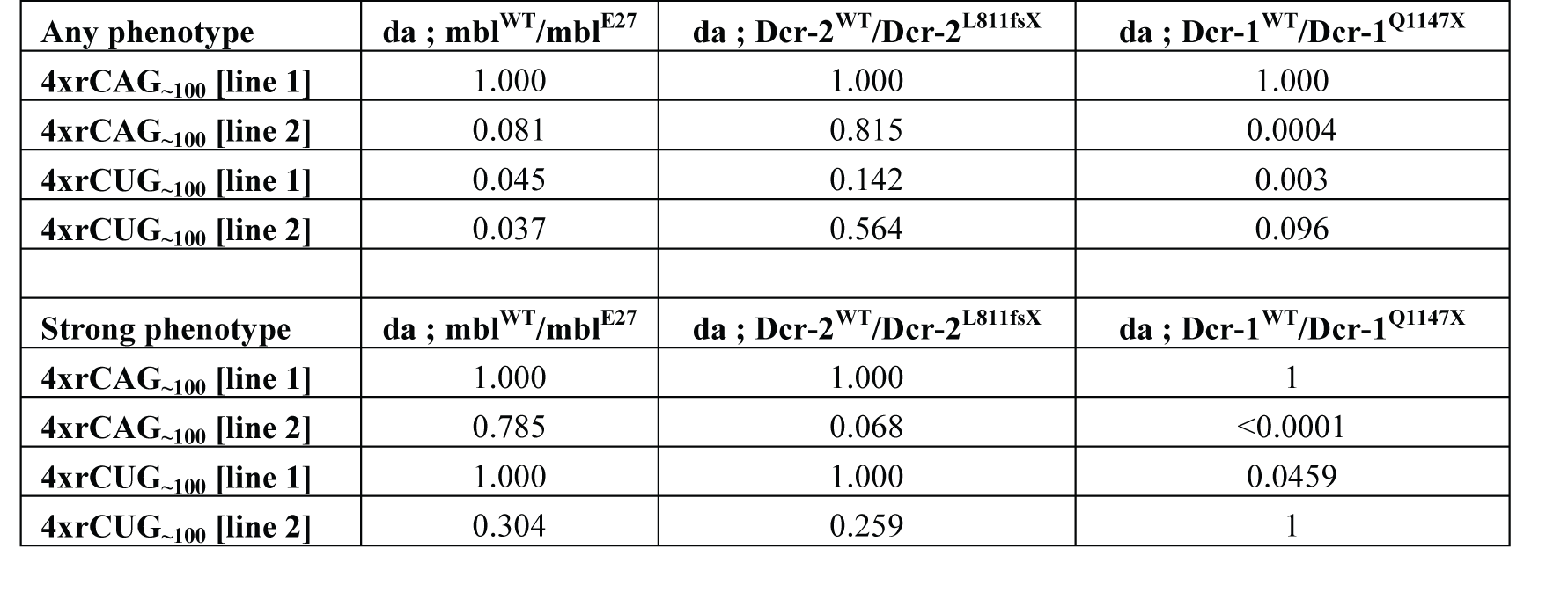

Supplement: Table S7 — Statistical comparison of tergite severity when different mutations are introduced. Tables shows p values from Fisher’s exact test comparing genotypes for the distribution between progeny with any phenotype (category 2, 3 and 4) and others, or between progeny with a strong phenotype (category 3 and 4) and others. In each case comparisons are made to the population expressing each repeat with da-GAL4 alone. (TIF) [file pone.0038516.s015.tif]
